# Structure of copper sites in zeolites examined by Fourier and wavelet transform analysis of EXAFS†

**DOI:** 10.1039/d0sc01472a

**Published:** 2020-05-01

**Authors:** Vitaly L. Sushkevich, Olga V. Safonova, Dennis Palagin, Mark A. Newton, Jeroen A. van Bokhoven

**Affiliations:** Laboratory for Catalysis and Sustainable Chemistry, Paul Scherrer Institut 5232 Villigen PSI Switzerland vitaly.sushkevich@psi.ch jeroen.vanbokhoven@chem.ethz.ch +41563103518; Laboratory for Operando Spectroscopy, Paul Scherrer Institut 5232 Villigen PSI Switzerland; Institute for Chemistry and Bioengineering, ETH Zurich Vladimir-Prelog-Weg 1 8093 Zurich Switzerland

## Abstract

Copper-exchanged zeolites are a class of redox-active materials that find application in the selective catalytic reduction of exhaust gases of diesel vehicles and, more recently, the selective oxidation of methane to methanol. However, the structure of the active copper-oxo species present in zeolites under oxidative environments is still a subject of debate. Herein, we make a comprehensive study of copper species in copper-exchanged zeolites with MOR, MFI, BEA, and FAU frameworks and for different Si/Al ratios and copper loadings using X-ray absorption spectroscopy. Only obtaining high quality EXAFS data, collected at large *k*-values and measured under cryogenic conditions, in combination with wavelet transform analysis enables the discrimination between the copper-oxo species having different structures. The zeolite topology strongly affects the copper speciation, ranging from monomeric copper species to copper-oxo clusters, hosted in zeolites of different topologies. In contrast, the variation of the Si/Al ratio or copper loading in mordenite does not lead to significant differences in XAS spectra, suggesting that a change, if any, in the structure of copper species in these materials is not distinguishable by EXAFS.

## Abstract 2

The structure of copper-oxo species hosted in zeolites of various topology has been examined using wavelet and Fourier transform analysis of Cu K-edge EXAFS spectra.

## Graphical abstract

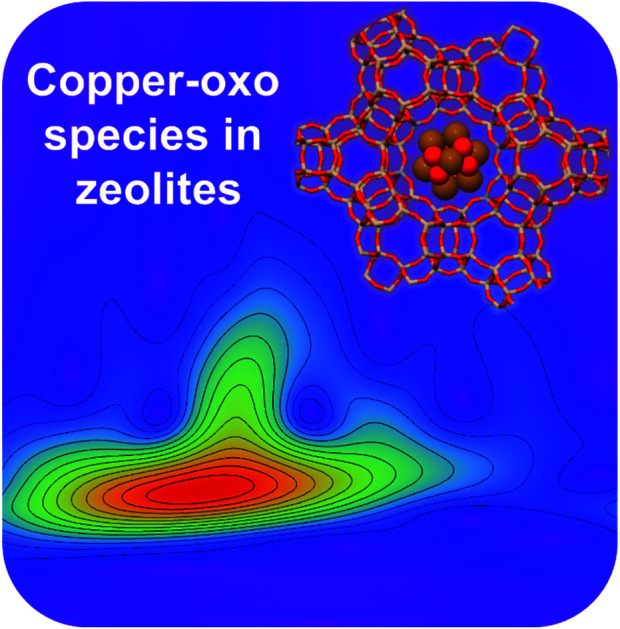

## 1. Introduction

Copper-exchanged zeolites have been in the focus of comprehensive studies for decades.^1–3^ From a material's point of view, they are excellent adsorbents for various nitrogen- and sulfur-containing compounds and can be used for waste water treatment.^4,5^ The catalysis community extensively exploits copper zeolites for the selective catalytic reduction (SCR) of NO_*x*_ in exhaust gases and for direct decomposition of NO and N_2_O.^6–9^ It has been recently shown that after pre-oxidation these systems can be used as highly efficient stoichiometric oxidants in the selective conversion of methane to methanol.^10–15^ In the light of these opportunities for application, the understanding of the structure of the copper species present within zeolites is of paramount importance.

The high activity of copper-containing zeolites in oxidation and reduction reactions is typically associated with the redox transformations of copper species, hosted in the zeolite's pore system. In general, the Cu^II^/Cu^I^ redox couple is involved in the reaction; the copper acts as a donor or acceptor of electrons and enables the conversion of the feed to the desired products.^16–24^ For the SCR of NO_*x*_, a mixture of Cu^II^ and Cu^I^ active species of varied structures is observed, with the relative fractions governed by the reaction conditions.^16–21^ Alternatively, for methane oxidation to methanol, the active species are represented exclusively by Cu^II^-oxo sites of different nuclearity, some of which may be converted to Cu^I^ upon contact with methane.^22–24^

The investigation of the species active in these particular reactions requires a method, which is element-selective and is able to follow the structure and electronic states of copper species. In this respect, X-ray absorption spectroscopy (XAS) represents an excellent choice due to its high sensitivity to the local environment, symmetry and the oxidation state of the elements of interest.^25,26^ Therefore, it is not surprising that a large fraction of the reports describing the performance of copper-exchanged zeolites utilize various variants of this method.^12–15,18,20–24,27–30^ Two separate techniques are mostly used: X-ray absorption near edge structure (XANES) and extended X-ray absorption fine structure (EXAFS). The first approach allows one to determine, and then follow, the oxidation state of copper in zeolites in the course of reaction and to access the aspects of the local symmetry of corresponding species.^25,26^ Due to the significantly different spectroscopic signatures of Cu^I^ and Cu^II^ species with various ligands, the analysis of the data is relatively straightforward and can be done by employing linear combination fitting or multivariate curve resolution methods.^15–18,21,23,31–33^ Hence, the fractions of different copper species can be extracted; time-resolved measurements enable the monitoring of the reaction kinetics and determination of the rate constants and apparent activation energies.^21,24,30^ However, the exact determination of the structure of copper species exclusively from a single XANES spectrum is challenging; DFT-assisted modelling represents a promising approach, while requiring more sampling and further development.^34,35^ Therefore, in the general case, the structural analysis of XANES relies on the comparison with the spectra of standard molecular compounds with known structures.

In contrast, EXAFS modelling is a robust, straightforward technique, and can be utilized on a routine basis for suitable materials, yielding coordination numbers and effective distances between atoms.^25,26^ It is element-specific and quantitative and can be applied under *in situ*/*operando* conditions.^25^ Unfortunately, the fitting of EXAFS is simple and highly effective mostly for highly ordered materials. Copper-exchanged zeolites do not meet this requirement due to the formation of a mixture of copper species in the pores, resulting in disordering.^2,15,18,24,28^ This leads to extremely small differences in the EXAFS spectra of different copper-exchanged zeolites measured in a short *k*-range, which therefore cannot provide any distinct structural information on the nature of multiple copper sites present in the system.^28^ According to UV-vis, resonant Raman, XAS, EPR and IR spectroscopies, Cu^II^-oxo species are believed to comprise copper dimers, such as bis-μ-oxo^10,27^ and mono-μ-oxo^11,36^ sites, trimers Cu_3_O_3_^2+^,^12^ and proximal monomers CuOH^+^.^15,37^ Moreover, all these sites are relatively similar from the EXAFS point of view due to the presence of similar scatterers in the first and second coordination spheres of the absorbing copper atom as well as overlapping contributions from silicon/aluminum and copper in the second shell. Hence, the existence of multiple copper-oxo species requires special care in both collecting EXAFS spectra and their comprehensive analysis.

In this respect, we report the analysis of EXAFS of Cu^II^-oxo species hosted in a series of oxygen-activated copper-exchanged zeolites with MOR, MFI, BEA, and FAU frameworks with different Si/Al ratios and copper loadings. We show that the contributions from Cu–Al(Si) and Cu–Cu scattering in the second coordination shell can be resolved by wavelet transform of the EXAFS spectrum. Notably, EXAFS spectra with a large *k*-range contain important information on the copper coordination related to Cu–Cu scattering pathways, suggesting the formation of different types of copper oxo species. Our results illustrate the power and point out the limitations of advanced EXAFS spectroscopy for copper-containing zeolites, which is of practical importance for the design and optimization of copper-based materials for environmental applications.

## 2. Experimental

### 2.1 Synthesis of materials

All samples were synthesized by conventional ion exchange in an aqueous solution of copper nitrate. Commercial zeolites, supplied by Zeolyst, namely MOR (CBV10A, CBV21A, CBV90A), MFI (CBV2314), BEA (CP814E), and FAU (CBV720), were used as the starting materials.

Prior to the copper ion exchange, all zeolites were converted to the ammonium form as described elsewhere.^31,38^ For complete ion exchange with copper, 10 g of each zeolite in the ammonium form were stirred in 1000 ml of a 0.05 M aqueous solution of copper nitrate (99%, Merck) at 323 K for 12 hours. The resulting sample was filtered, washed with 1 l of deionized water and dried at 393 K for 1 h. The ion exchange was repeated two times. The final material was calcined at 773 K for 3 h in a flow of dry synthetic air.

For the partial ion exchange of mordenite (CBV10A) with copper, aqueous solutions of copper nitrate with concentrations ranging from 0.005 to 0.05 M were used.^38^ The copper content as well as the Si : Al : Na ratio were determined by inductively coupled plasma mass spectrometry (ICP-MS) with an Agilent 77009 ICPMS instrument after complete digestion of the samples in 2 M hydrofluoric acid. Table 1 summarizes the elemental composition of the synthesized materials. The bulk properties are explicitly reported in our previous studies.^15,31,38^ All synthesized materials were denoted as Cu(*x*)ZEO(*y*), where “ZEO” represents the zeolite framework type of the material, and “*x*” and “*y*” correspond to the copper loading in wt% and the Si/Al ratio, respectively.

**Table 1:** Characteristics of the materials

Sample	Chemical composition			
	Cu, wt%	Na, wt%	Si/Al	Cu/Al
Cu(3.4)MOR(10)	3.44	<0.01	10.5	0.386
Cu(4.0)MFI(12)	4.02	<0.01	11.6	0.534
Cu(2.8)BEA(12)	2.83	<0.01	12.4	0.402
Cu(2.7)FAU(15)	2.70	<0.01	14.8	0.395
Cu(4.4)MOR(6)	4.36	1.22	6.5	0.377
Cu(1.2)MOR(46)	1.22	<0.01	46	0.603
Cu(3.5)MOR(6)	3.52	1.93	6.5	0.309
Cu(3.2)MOR(6)	3.28	2.34	6.5	0.262
Cu(2.5)MOR(6)	2.54	2.73	6.5	0.210
Cu(1.7)MOR(6)	1.74	3.38	6.5	0.144

### 2.2 Preparation for XAS measurements

All samples were placed into thin-wall (10 μm) quartz capillaries with an external diameter of 1.5 mm, which were selected based on the copper loading in the studied samples. The capillaries were attached to a vacuum rig and activated as follows: evacuation at 673 K for 2 h, oxidation in 300 torr of oxygen at 673 K for 1 h with subsequent evacuation of the gas and traces of water at 573 K for 1 h to avoid significant auto-reduction.^38^*In situ* oxidation was performed to exclude the reduction of Cu^II^ species by traces of organic compounds, which might be present in the zeolite after the synthesis or adsorbed from air.^39^ After activation, the capillaries were cooled down to ambient temperature and sealed-off under vacuum using a propane torch. The sealed capillaries were stored under ambient conditions in a dark place.

### 2.3 XAS measurements

Cu K-edge X-ray absorption spectra were recorded at the SuperXAS beam line, Swiss Light Source, Switzerland. The polychromatic beam was collimated by a silicon-coated mirror at 2.5 mrad (which reduces higher-order harmonics) and subsequently monochromatized by a Si(111) channel-cut monochromator, which allowed data collection in quick-scanning extended X-ray absorption fine structure spectroscopy mode (QEXAFS) at 1 Hz.^40^ The amplitude of monochromator oscillations was selected to obtain EXAFS spectra within an energy range between 8800 and 10 700 eV. The monochromatic beam was focused by a toroidal rhodium coated mirror at 2.5 mrad. Spectra were collected in transmission mode using nitrogen-filled ionization chamber detectors. A copper reference foil mounted between the second and the third ionization chamber was measured simultaneously for absolute energy calibration. Each sample was placed between the first and second ionization chambers, cooled down to 130 K with a cold nitrogen vapor jet (Cryostream, Oxford Cryosystems) and measured to obtain 1200 single spectra for each sample. For comparison, the spectrum of bulk copper(ii) oxide was acquired. Then, QEXAFS spectra were averaged to achieve a high signal-to-noise ratio using JAQ software.^40^ Normalization and background subtraction were carried out using Athena software from the Demeter package.^41^

### 2.4 FT EXAFS analysis and fitting

The Fourier transform (FT) fitting of the EXAFS spectra of copper-exchanged zeolites was carried out using Artemis software.^41^ The phase and amplitude were calculated using the FEFF6 code.^42,43^ For the FT, *k*^3^-weighting was used in the *k* range of 3.0–16.0 Å^−1^, with fitting in *R*-space within the Δ*R* = 1.0–3.0 Å region. The apodization function was shown to have no significant effect on the results of FT and further fitting (Fig. S1†). For the copper-exchanged BEA and FAU the Δ*R* was extended to 1.0–3.2 Å due to the presence of multiple peaks in the second coordination shell. The amplitude reduction factor was estimated by the first-shell fitting of the copper foil standard resulting in a value of 0.90, which was used throughout the study.

A periodic structure of dicopper mono-μ-oxo species located in an 8-membered ring of mordenite, optimized with density functional theory (DFT) (see the Computational Details section in the ESI†), was used as the initial model for the fitting of Cu–O, Cu–Al(Si) and Cu–Cu single scattering paths in the spectra of CuMOR and CuMFI materials. For the modelling of EXAFS spectra of CuBEA and CuFAU, the copper oxide nanocluster comprising eight copper atoms was placed in the supercage of faujasite and optimized with DFT. The optimized geometries have been adopted as the initial input for the FEFF calculations. The experimental EXAFS spectra were fitted including only single-scattering paths in the fitting model, which represent the dominant contribution in the *R*-space interval of interest (1.0–3.2 Å). Multiple scattering pathways were excluded based on the results of the fitting of *k*^0^ and *k*^1^-weighted data (Fig. S2†), which showed the absence of additional contributions in the *R*-space between 1.0 and 3.0 Å. The same conclusion was derived from fitting of the EXAFS of standard copper(ii) oxide with single scattering paths (Fig. S3, Table S1†). The number of parameters employed in the fits varied from 10 to 12, depending on the different input geometries. The quality of the fits was quantified by the *R*-factor and *χ*^2^ parameters.

### 2.5 WT EXAFS analysis

Wavelet transform (WT) analysis is a powerful technique, coming from the signal processing field, which has attracted significant attention in the field of X-ray absorption spectroscopy.^44–48^ This is due to the possibility that arises from this approach to resolve overlapping contributions coming from different neighbor atoms. If two or more groups of different atoms are localized at close distances around the absorbing atom, their contributions in the direct *R* space overlap, making the classical FT analysis of the EXAFS spectrum rather challenging. However, if the scattering atoms have sufficiently different backscattering factors, it is possible to observe the localization of the curved-wave harmonic contributions, coming from the different scatters in *k*-space. The backscattering factor *F*(*k*) strongly depends on the atomic number, *Z*.^49^ With that, heavy atoms are localized at higher wavenumbers in the EXAFS spectrum compared to the lighter ones. This fundamental issue constitutes the basis for the WT analysis that allows signal discrimination on the basis of a two-dimensional representation of the EXAFS spectrum with a simultaneous signal localization in *k* and *R* space.^45–47^ However, it is important to notice that the Heisenberg uncertainty principle applies for wavelet analysis: equally high resolution in both *k*- and *R*-space is not achievable.^44^ This emphasizes the importance of the selection of the correct mother function, which might be different for different types of analyses. In addition, the possibility of constructive and destructive interference of the backscattered waves should not be neglected since it can lead to the appearance of several lobes in the WT transform corresponding to one group of atoms with identical *Z* (Fig. S4†).

For the wavelet transform, *k*^3^-weighted EXAFS spectra were used. We employed a Morlet wavelet mother function for the WT transform of all spectra within the *k*-range of 1–16 Å^−1^.^48^ The selection of this wavelet was governed by the fast oscillatory part localized in a Gaussian envelope, making its real and imaginary parts similar to an EXAFS spectrum. Continuous wavelet transform was carried out for 128 data points using a built-in function in Mathematica software,^50^ with subsequent visualization as 2D counterplot projection of a 3D graph.

## 3. Results

### 3.1 Cu K-edge XANES of copper-exchanged zeolites

**Fig. 1:**
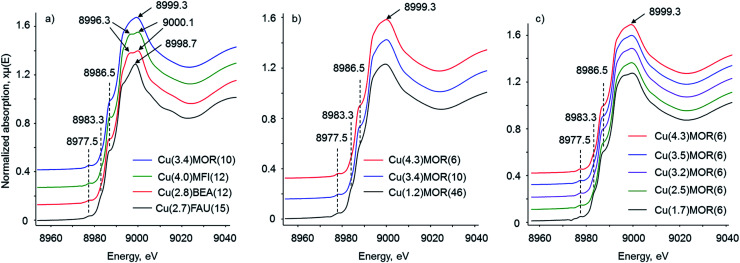
Cu K-edge XANES spectra of copper-exchanged zeolites of (a) different topologies, (b) Si/Al ratios and (c) copper loadings collected at 130 K. The samples were pre-oxidized in 300 torr of oxygen for 1 h at 673 K with subsequent evacuation at 573 K for 1 h.

Three sets of copper-containing zeolites with (i) different zeolite topologies, (ii) different Si/Al ratios (for mordenite samples), and (iii) different copper loadings (for mordenite samples with Si/Al = 6) were investigated. Table 1 gives the elemental composition of the prepared materials. First, Cu K-edge XANES spectra acquired after the activation of the samples in oxygen with subsequent evacuation at 573 K were analyzed (Fig. 1). All spectra demonstrate the features typical of the Cu^II^ species: the shoulder at 8986.5 eV together with the peak at 9000 eV corresponds to 1s → 4p transition, while the weak pre-peak at 8977.5 eV is due to the dipole-forbidden 1s → 3d transition.^15,20–26,28–31^ Apart from Cu^II^ species, the presence of a small number of Cu^I^ sites is also evidenced by the observation of a small shoulder at 8983.3 eV due to 1s → 4p conversion. These Cu^I^ species originate from the auto-reduction of Cu^II^ during thermal treatment of copper-exchanged zeolites under vacuum.^38,51,52^ Linear combination fitting analysis estimates the amount of Cu^I^ which is below 3% for mordenite samples and below 6% for CuBEA and CuFAU, pointing to the high resistance towards auto-reduction at 573 K, in line with previous reports (Fig. S5†).^15,29,38^

Notably, the relative intensity and the position of the signals in the 8970–8990 eV energy range does not vary in the spectra of all studied samples of different topologies, Si/Al ratios and copper contents. In contrast, for the copper-containing zeolite of different topologies, the features located within the 8990–9020 eV range differ significantly (Fig. 1a). Hence, the spectrum of Cu(3.4)MOR(10) shows the presence of one broad asymmetric peak with the maximum located at 8999.3 eV. Cu(4.0)MFI(12) and Cu(2.8)BEA(12) reveal similar spectra with at least two resolved peaks centered at 8996.3 and 9000.1 eV. Finally, the spectrum of Cu(2.7)FAU(15) demonstrates a sharp resolved signal at 8998.7 eV with two visible bumps at about 9006 and 9016 eV. The last two signals are also observable in the spectrum of the Cu(2.8)BEA(12) sample; however, they have much lower intensity. For most of the copper-exchanged mordenite samples, the variation of the Si/Al ratio or copper loading (Fig. 1b and c) does not result in detectable changes in Cu K-edge XANES spectra. Only the Cu(1.7)MOR(6) sample demonstrates a slight change in the shape of the white line, which might be associated with a higher amount of Cu^I^ species formed *via* auto-reduction, as compared to the other CuMOR samples.

### 3.2 Cu K-edge EXAFS of the copper-exchanged zeolites

**Fig. 2:**
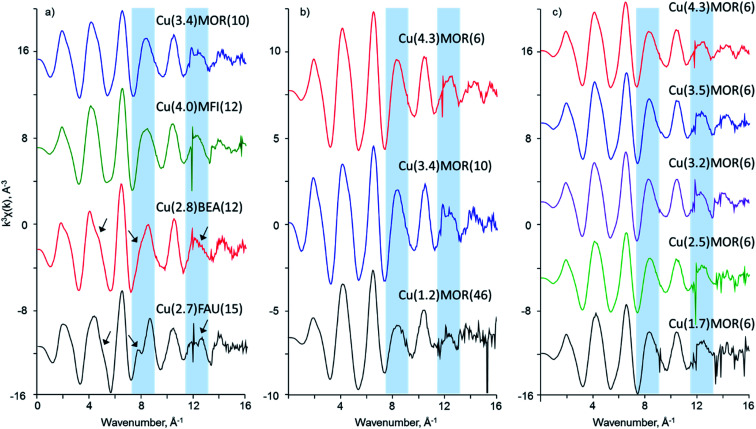
*k* ^3^-weighted *χ*(*k*) EXAFS data for activated copper-exchanged zeolites acquired at 130 K. Blue sectors indicate the regions of the largest variation observed for the zeolites of different topologies, indicating the formation of different copper species.

In further analysis of XAS data, EXAFS spectra were carefully examined. Fig. 2 shows the *k*^3^-weighted *χ*(*k*) data for oxygen-activated copper-exchanged zeolites acquired at 130 K. The first set of spectra, due to the copper-exchanged zeolites with different topologies, reveals a pronounced difference in the EXAFS spectra at frequencies around 8 and 12 Å^−1^. Cu(2.8)BEA(12) shows the presence of shoulders in these regions; in the case of Cu(2.7)FAU(15), these shoulders become better resolved. Moreover, careful analysis of the signals around 5 Å^−1^ reveals a considerable change of the shape of EXAFS spectra. None of these features were observed in the EXAFS spectra of Cu(3.4)MOR(10) and Cu(4.0)MFI(12).

In contrast, the copper-exchanged mordenites with different Si/Al ratios and copper loadings give very similar EXAFS spectra in *k*-space (Fig. 2b and c). No significant differences were observed in 5, 8 and 12 Å^−1^ regions upon variation of the chemical composition of the materials. The only exception is Cu(1.2)MOR(46), which possesses a lower intensity of the EXAFS signals in the 8–16 Å^−1^ range. However, due to its low copper content, the signal-to-noise ratio is rather poor at high frequencies, which complicates the detailed assessment.

**Fig. 3:**
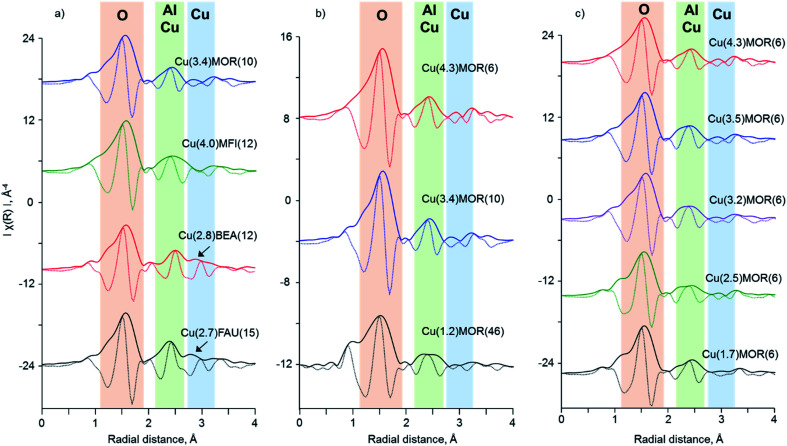
Phase-uncorrected FT EXAFS spectra obtained from *k*^3^-weighted *χ*(*k*) data in the range of 3–16 Å^−1^. Solid and dotted lines correspond to the magnitude and real part of the Fourier transform, respectively. Colored sectors denote the main contributors to the scattering in the selected regions: oxygen (red), aluminum, silicon or copper (green) and exclusively copper (blue). The arrows indicate the presence of additional contributions in the second coordination shell for Cu(2.8)BEA(12) and Cu(2.7)FAU(15).

Fig. 3 gives the Fourier transform of *k*^3^-weighted EXAFS spectra within the 3–16 Å^−1^ range. After FT, the prominent difference observed in *k*-space EXAFS spectra of copper-exchanged zeolites of different topologies becomes more resolved in *R*-space (Fig. 3a). In FT EXAFS of Cu(3.4)MOR(10) and Cu(4.0)MFI(12) two peaks corresponding to the first and second coordination spheres are visible at the phase-uncorrected radial distances of about 1.5 and 2.3 Å.^2,12,14,21,22,24,27,28,33^ The first peak originates from the oxygen atoms surrounding the copper atom.^2,12,14,21,22,24,27,28,33^ The second peak is typically associated with the superposition of contributions from the framework aluminum or silicon atoms and extra-framework copper atoms.^2,12,24–26,33^ In addition to these peaks, the FT EXAFS of Cu(2.8)BEA(12) and Cu(2.7)FAU(15) show the presence of an additional peak in the second coordination sphere at about 2.8 Å (phase-uncorrected). This observation indicates the presence of other types of copper species in copper-exchanged BEA and FAU.

Similarly to *k*-space data, the FT EXAFS spectra of copper-exchanged mordenite do not show significant changes upon variation of the Si/Al ratio and copper content. Only a single peak is observed in the second coordination sphere at 2.3 Å due to aluminum, silicon or copper atoms, with no contribution at higher frequencies.

### 3.3 Cu K-edge EXAFS of copper(ii) oxide

**Fig. 4:**
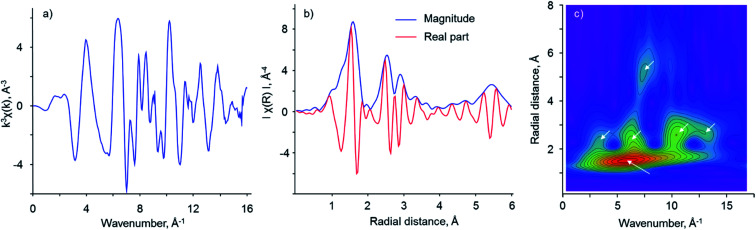
(a) *k*^3^-weighted *χ*(*k*) EXAFS data for CuO; (b) corresponding phase-uncorrected FT EXAFS spectra and (c) 2D plots of WT EXAFS for CuO.

The detection of the second contribution at 2.8 Å in the second shell of FT EXAFS spectra for Cu(2.8)BEA(12) and Cu(2.7)FAU(15) indicates the possible formation of a new type of copper-oxo species. In this respect, the comparison with the bulk copper(ii) oxide of the tenorite structural type helps in the assignment of this peak. Fig. 4 reports the corresponding EXAFS data of this standard compound. The spectrum in *k*-space differs significantly from the ones collected for all copper-exchanged zeolites: at about 8–10 Å high frequency oscillation is present, which is convoluted with the main low frequency one. This makes the direct comparison of the spectrum of copper oxide in the *k*-space with that of copper zeolites difficult. However, it should be noted that at about 12 Å a combination of features similar to Cu(2.7)FAU(15) is also observed for CuO.

Resembling copper-exchanged zeolites, FT EXAFS data for copper(ii) oxide (Fig. 4b) show the presence of two well-resolved peaks due to the first and the second coordination sphere. Moreover, the second coordination shell peak splits into two separate contributions at 2.5 and 2.9 Å, which is the result of destructive interference of the scattering waves from three copper atoms located at 2.88, 3.07 and 3.16 Å apart from the absorbing atom. Additionally, a broad signal centered at 5.5 Å is visible. To correlate these FT peaks with *k*-space data, wavelet transform was employed (Fig. 4c). Note that all WT EXAFS are not phase-corrected, which results in about 0.4 Å shift with respect to the actual radial distances. The highest intensity belongs to the lobe centered at (6 Å^−1^; 1.5 Å), corresponding to the oxygen atoms surrounding the central copper atom. At higher radial distances within the range of 2–3 Å, several resolved lobes are located at approximately (3.5 Å^−1^; 2.3 Å), (6.5 Å^−1^; 2.5 Å), (10.5 Å^−1^; 2.8 Å) and (13.0 Å^−1^; 2.7 Å). To assign them, we use the dependence of the backscattering factor *F*(*k*) on the atomic number *Z*, which indicates that the heavy atoms, having large values of *F*(*k*), are localized at higher frequencies in *k*-space than lighter atoms and *vice versa* (Fig. S6†). Therefore, the lobe at (3.5 Å^−1^; 2.3 Å) is due to the oxygen atom, which appears at 2.77 Å in the tenorite structure. The remaining three lobes, having higher *k*-values, are due to the copper scattering atoms, in line with FT EXAFS data. The splitting into several lobes is associated with the destructive interference of backscattered waves, resulting in several maxima for the magnitude of the signal (Fig. S4†). Finally, the broad lobe centered at (7.5^−1^ Å; 5.2 Å) and spanning to the region of higher *k*-values can be clearly linked with the signal at 5.5 Å in the FT EXAFS of copper(ii) oxide. High *k*-values for this signal attribute it to the copper atoms, participating in backscattering, including multiple scattering paths. Note that the WT of *k*^2^-weighted EXAFS spectrum preserves its structure pointing to a minimal effect of noise at high *k* values (Fig. S7†).

### 3.4 Cu K-edge WT EXAFS of copper-exchanged zeolites

**Fig. 5:**
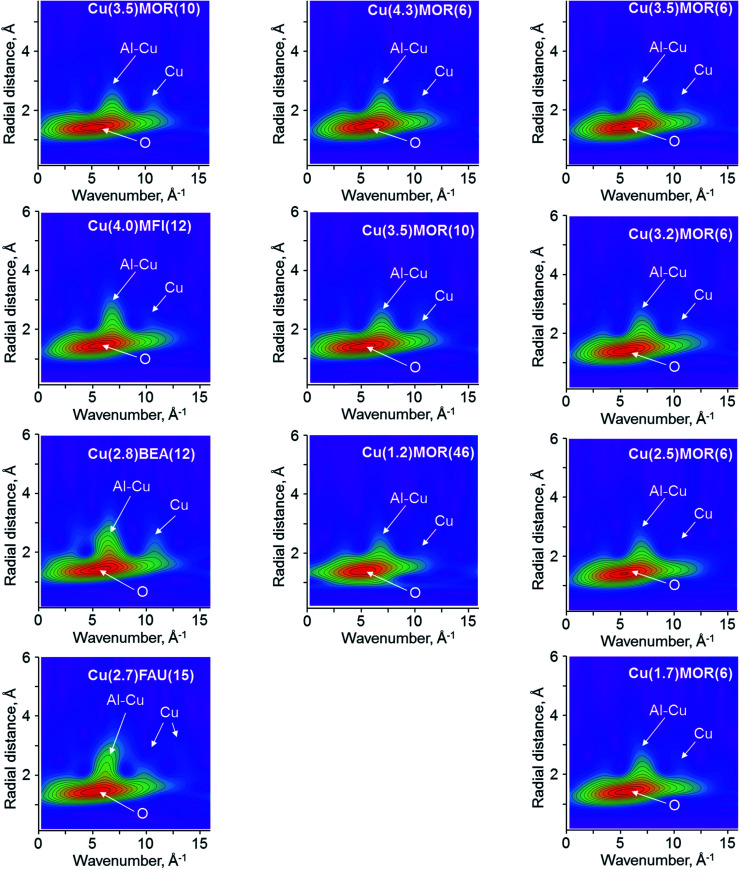
Experimental 2D WT EXAFS plots for *χ*(*k*) EXAFS data collected for copper-exchanged zeolites at 130 K. The arrows indicate the main scattering contributions to the corresponding lobes in WT spectra. Left column compares the copper-exchanged zeolites with different topologies, the column in the center corresponds to the CuMOR with different Si/Al ratios and the column on the right shows the CuMOR with different copper loadings.

Fig. 5 shows the results of wavelet transform applied to the EXAFS spectra of the copper-exchanged zeolites. First, we analyze the set of the copper-exchanged samples with different zeolite topologies. Cu(3.4)MOR(10) shows the most intense signal centered at (5.0 Å^−1^; 1.5 Å), assigned to the first shell oxygen atoms of the zeolite framework, which are coordinated to the copper. At higher *k*-values, three lobes are resolved at about (3.5 Å^−1^; 2.4 Å), (7.0 Å^−1^; 2.4 Å) and (11.0 Å^−1^; 2.5 Å), corresponding to the second coordination sphere. According to the low *k* value, the first signal originates from framework or extra-framework oxygen atoms, while the last peak with high *k* is due to copper atoms. The asymmetric lobe in the middle at (7.0 Å^−1^; 2.4 Å) most probably comprises several contributions: first coming from aluminum or silicon atoms of the framework and second from copper atoms, as observed for copper(ii) oxide.

WT EXAFS for the Cu(4.0)MFI(12) material differs insignificantly from that of Cu(3.4)MOR(10), as the same combination of signals is present (Fig. 5). The only detectable change is associated with the increase of the intensity and broadening of the lobe at (7.0 Å^−1^; 2.4 Å), which sums the contributions from aluminum, silicon and copper scatters. For Cu(2.8)BEA(12), a more pronounced change of this signal is visible; it becomes more asymmetric and intense, spanning up to ∼3.5 Å in *R*-space. Together with that, the signal at (11.0 Å^−1^; 2.5 Å) demonstrates a higher intensity as compared to the one of Cu(3.4)MOR(10) and Cu(4.0)MFI(12). The WT EXAFS spectrum for Cu(2.7)FAU(15) reveals a substantially different picture with respect to the previously discussed copper-exchanged zeolites: the maximal intensity of the lobe due to the superposition of contributions from aluminum, silicon and copper shifts to a (6.0 Å^−1^; 2.5 Å) position. Simultaneously, two lobes at higher *k*-values appear at (11.0 Å^−1^; 2.6 Å) and (13.0 Å^−1^; 2.8 Å), corresponding to the scattering from copper atoms. Such a significant difference indicates the presence of copper-oxo species, which might have multiple copper atoms in the second coordination shell.

Varying the silicon to aluminum ratio in copper-exchanged mordenite does not lead to the appearance or disappearance of the signals in WT EXAFS (Fig. 5). Four major lobes at (5.0 Å^−1^; 1.5 Å), (3.5 Å^−1^; 2.4 Å), (7.0 Å^−1^; 2.4 Å) and (11.0 Å^−1^; 2.5 Å), assigned to oxygen, aluminum, silicon or copper scatters, are well-resolved in the spectra. Notably, the intensity of the latter due to copper atoms in the second coordination sphere is significantly lower for the Cu(1.2)MOR(46) sample, while for other MOR materials the intensity is preserved. This points to a small contribution of copper-oxo oligomeric species, comprising two or more copper atoms in their structure, and confirms the preferential formation of copper monomers, as was previously suggested based on the results of IR spectroscopy of adsorbed nitrogen monoxide and *in situ* XPS.^15,53^

In the case of different copper loadings in mordenite, the obtained WT EXAFS are extremely similar to the one observed for Cu(4.3)MOR(6). Even a significant decrease of copper loading from 4.3 to 1.7 wt% does not lead to the disappearance of the lobe at (11.0 Å^−1^; 2.5 Å) due to copper scatters, pointing to the formation of copper-oxo oligomers in all CuMOR(6) samples.

## 4. Discussion

The analysis of the results of XANES, FT EXAFS and WT EXAFS shows that each approach provides important information for the assessment of the nature of copper-oxo species stabilized in zeolites. This allows a clarification of the effects of the zeolite type, Si/Al ratio, and copper loading on the structure of copper after activation in an oxidative environment.^54^

The analysis of XAS data for the copper-exchanged zeolites of different topologies reveals the presence of significantly different copper-oxo species in these materials (Fig S8–S11†). For quantitative analysis, we performed the fitting of EXAFS data, and Table 2 summarizes the obtained results. The fitting of the first coordination shell is straightforward due to the exclusive contribution of oxygen scattering atoms to the peak at about 1.95 Å. Measured coordination numbers (CN) for copper, surrounded by oxygen, are between 3.5 and 3.9. In most previous studies, coordination numbers close to three are reported;^32,33,55–58^ however, in several papers a CN of four was observed.^28,59–62^ Our values fall in this range and, considering the relative accuracy of EXAFS in establishing coordination numbers, correlate with previous reports. The presence of a small amount of Cu^I^ species (Fig. 1), formed during the activation of the samples and typically possessing a CN of two, can also contribute to the total coordination of copper, measured by EXAFS.

**Table 2:** Best-fit parameters optimized by EXAFS fits of the *k*^3^-weighted spectrum of activated copper-exchanged zeolites of different topologies employing two DFT-optimized geometries for dicopper mono-μ-oxo sites and copper clusters as the starting guess. The fit was performed in *R*-space in the range of 1.0–3.2 Å, employing the *k*-range of 3.0–16.0 Å^−1^ for the FT, resulting in a number of independent points *N*_ind_ > 16

Parameter	Scatter	Cu(3.4)MOR(10)	Cu(4.0)MFI(12)	Cu(2.8)BEA(12)	Cu(2.7)FAU(15)
Δ*E*, eV		+2.9(7)	+2.3(6)	0.3(9)	0.1(9)
*N*_O_	O	3.7(2)	3.9(2)	3.7(2)	3.5(3)
*R*(Cu–O), Å		1.944(3)	1.944(3)	1.943(4)	1.959(5)
Debye–Waller factor, 10^−3^ Å^2^		6.3(3)	6.0(3)	5.5(4)	6.1(6)
*N*_Al_	Al or Si	1.7(5)	1.1(3)	1.2(4)	1.1(4)
*R*(Cu–Al, Si), Å		2.70(1)	2.67(1)	2.70(2)	2.77(8)
Debye–Waller factor, 10^−3^ Å^2^		10.8(38)	6.7(20)	4.7(20)	11.7(22)
*N*_Cu_	Cu_1_	0.3(1)	1.1(4)	2.3(9)	1.7(9)
*R*(Cu–Cu_1_), Å		2.841(16)	2.904(8)	2.888(3)	2.938(2)
Debye–Waller factor, 10^−3^ Å^2^		5.0(21)	9.4(27)	10.0(33)^a^	6.9(24)^a^
*N*_Cu_	Cu_2_			2.6(9)	1.8(9)
*R*(Cu–Cu_2_), Å				3.05(1)	3.08(1)
Debye–Waller factor, 10^−3^ Å^2^				10.0(33)^a^	6.9(24)^a^
*R*-factor		0.0029	0.0024	0.0054	0.0066
*χ* ^2^-parameter		187	23	134	116

a Fixed to be equal in the fit. Both scatters are of the same nature (copper atoms); therefore, their Debye–Waller factors are assumed to be similar.

The fitting of the second coordination shell is more challenging. First, EXAFS cannot distinguish between the scattering of aluminum and silicon atoms due to their similar atomic numbers.^32,33^ Therefore, for the sake of clarity, below we designate their contributions as “Al/Si”, which would virtually mean that both Al and Si atoms are contributing to the particular EXAFS pattern. Using DFT-assisted EXAFS fitting it was demonstrated that employing both aluminum and copper scatterers allows for a significant improvement of the quality of the fit (Fig. S12†).^12,14,24,32,33^ Previous investigations of theoretically modeled EXAFS spectra showed the principal possibility to resolve Cu–Cu scattering pathways in the second coordination shell using WT.^44,48^ In this respect, our wavelet analysis provides unambiguous evidence, which enables the discrimination of the different scatters contributing to the peak at about 2.3 Å in FT EXAFS. The presence of two separate lobes at about (7.0 Å^−1^; 2.4 Å) and (11.0 Å^−1^; 2.5 Å) in WT EXAFS for Cu(3.4)MOR(10) and Cu(4.0)MFI(12) indicates the existence of both Cu–Al/Si and Cu–Cu paths (Fig. 5). Having these data, we used a DFT-optimized model of dicopper mono-μ-oxo sites, which possesses the neighboring copper and aluminum atoms at a distance of about 2.7 Å from the central copper as the initial guess for the fitting of the second coordination shell (Table 2). Cross-validation of WT EXAFS by the fitting of FT EXAFS data returned unsatisfactory fits for exclusively aluminum or copper scatters, which is in line with previous reports (Fig S12 and S13†).

The fitted Cu–Al and Cu–Cu distances are slightly different: in the case of Cu(4.0)MFI(12) the aluminum atom is located closer (2.67 Å) and the copper atom is located farther (2.90 Å) from the central scatter, with respect to the values calculated for Cu(3.4)MOR(10). This indicates a different geometry and, in particular, the Cu–O–Cu bond angle of copper-oxo species governed by the pore structure of zeolites, in line with previous DFT studies.^63,64^ The second shell coordination number for Al/Si in Cu(3.4)MOR(10) is 1.7, while for Cu(4.0)MFI(12) it is lower and has a value of 1.1. Such a low CN for Al/Si in the second shell in the case of copper-exchanged MFI comes together with a relatively high CN for copper, estimated to be close to 1 (Table 2). In contrast, Cu(3.4)MOR(10) gives a coordination number for copper of only 0.25. Summarizing these data, copper-exchanged MFI on average has at least one copper and one aluminum/silicon atom in the second coordination shell, which agrees with the simplest model of dicopper mono-μ-oxo sites. The formation of dicopper bis-μ-oxo sites and tricopper oxo species or a mixture of thereof cannot be excluded based on such analysis. However, comparing this sample with Cu(3.4)MOR(10), which has a significantly smaller contribution of copper in the second shell, it can be concluded that the fraction of multinuclear copper-oxo sites is considerably lower in copper-exchanged mordenite with respect to copper-exchanged MFI. For Cu(3.4)MOR(10), the formation of a large fraction of copper monomeric sites, such as Cu^2+^and CuOH^+^, explains the low CN for copper in the second coordination shell, while for Cu(4.0)MFI(12) the presence of dicopper mono-μ-oxo sites has already been independently demonstrated by UV-vis and Raman spectroscopy.^65^ This discussion is in line with WT EXAFS spectra of these samples, showing different intensities of the lobe at (11.0 Å^−1^; 2.5 Å) due to the copper scattering atoms (Fig. 5). The noted difference is coherent with the changes in XANES spectra (Fig. 1a), which shows different structures of the white line for these two samples, hence confirming the presence of various copper-oxo species. The fitted Debye–Waller factors appear to be relatively high for the spectra collected under cryogenic conditions. We associate this observation with the structural rather than thermal disorder of the copper species.

Two other copper-exchanged zeolites, BEA and FAU, revealed the presence of a splitting of the peak in the second coordination sphere in FT EXAFS (Fig. 3a, S10 and S11†). Moreover, WT EXAFS shows at least three separate contributions to this peak, coming from the signals centered at 7.0, 11.0 and 13.0 Å^−1^ in *k*-space (Fig. 5). Comparing these patterns with the one obtained for bulk copper oxide (Fig. 4), we conclude that two distinct copper atoms are contributing to the second shell together with framework aluminum atoms. Therefore, we used a copper oxide cluster, having several different Cu–Cu distances, accommodated in FAU zeolite, as the starting model for the fitting of FT EXAFS.

Table 2 gives the results of the fitting for Cu(2.8)BEA(12) and Cu(2.7)FAU(15) with two distinct copper atoms contributing to the second shell. An aluminum or silicon atom is located ∼2.70 Å away from the central copper atom and has a coordination number close to 1; the distance and CN are similar to those observed for copper-exchanged MFI. The fitted Cu–Cu_1_ and Cu–Cu_2_ distances for Cu(2.8)BEA(12) are 2.89 and 3.05 Å, which are slightly lower than those for Cu(2.7)FAU(15) (2.94 and 3.08 Å, respectively). This difference is within the accuracy of the EXAFS method and virtually suggests similar distances for Cu–Cu in both Cu(2.8)BEA(12) and Cu(2.7)FAU(15). The coordination numbers for both copper scatters are different: for copper-exchanged BEA CNs are close to ∼2.6, while Cu(2.7)FAU(15) gives a value of ∼1.7. The average coordination numbers suggest the formation of on average larger copper-oxo clusters in copper-exchanged BEA compared to FAU. However, the CN of copper is still considerably lower than that in copper oxide with a tenorite structure, where the CN is equal to four. Also, the absence of high frequency oscillations within the range of 7–10 Å^−1^ in *k*-space and corresponding to the peak at about 5.5 Å in FT and WT EXAFS excludes the formation of very large copper oxide nanoparticles (Fig. 2a, 3a and 5). The formation of copper-oxo clusters is also in line with XANES, which in the case of Cu(2.7)FAU(15) shows a sharp peak at 8998.7 eV (Fig. 1a). A very similar signal centered at 8998.0 eV is present in the XANES spectrum of the copper(ii) oxide standard (Fig. S14†), hence suggesting the possible existence of copper-oxo species with multiple Cu–Cu contributions in Cu(2.8)BEA(12) and Cu(2.7)FAU(15). Notably, for Cu(3.4)MOR(10), which showed a very small contribution of copper to the second coordination shell, the white line peak is broad and shifted to a higher energy of 8999.3 eV (Fig. 1a). Therefore, we speculate that the position and the shape of the features in the XANES spectrum might be sensitive to the copper coordination and can be correlated with the nature of copper-oxo species hosted in oxygen-activated zeolites. However, more research is required in this direction.

Summarizing, the results of FT EXAFS fitting confirm the stabilization of completely different copper-oxo species in zeolites of different topologies and pore sizes. In copper-exchanged mordenite, the contribution from copper in the second coordination shell is not very pronounced which indicates the formation of a very high fraction of copper monomeric species. The fitted EXAFS coordination number of CuMFI is in agreement with the presence of dicopper species; however, other possibilities cannot be refuted. Copper-exchanged BEA and FAU show a high fraction of copper-oxo clusters of a very small size. This property to stabilize clusters might be associated with the size of the 12MR pores for BEA and the existence of the supercage in FAU.

We also considered the possibility that all copper-exchanged zeolites including the ones of different topologies have exactly the same copper-oxo species, but differ by the degree of static disorder. Practically, stronger disordering would lead to broader peaks in FT EXAFS, including the second coordination shell. If true, the models used for copper-exchanged faujasite and comprising two different Cu–Cu scattering pathways should be able to fit CuMOR and CuMFI spectra with only higher Debye–Waller factors, while other parameters should persist. To verify this hypothesis we performed the corresponding fits in *k*^3^-space. However, the obtained results showed significant misfits in the second coordination shell for both copper-exchanged MOR and MFI, together with worse statistical parameters as compared to the initial models (Table S2, Fig S15 and S16†). This points out that the difference observed in EXAFS spectra cannot be associated with the variation of static disordering only and indicates the presence of different copper-oxo species.

Next, the FT EXAFS spectra of copper-exchanged mordenite samples with different Si/Al ratios were fitted (Fig S8, S17 and S18†). Table 3 reports the obtained interatomic distances, coordination numbers and Debye–Waller factors. The increase of the Si/Al ratio from 6 to 10 does not lead to any considerable difference in the fits: all extracted parameters stay virtually the same. In contrast, a further decrease of the aluminum content to a Si/Al ratio of 46 in the parent mordenite results in significantly different fitting parameters for Al/Si atoms in the second shell: the coordination number decreases to 0.5 with the Cu–Al distance decreased to 2.64 Å as compared to Cu(4.3)MOR(6). Surprisingly, in spite of low probability to form multinuclear copper species in zeolite with high Si/Al, the fitted copper coordination number does not experience any change and is estimated at about 0.3, indicating the presence of Cu–Cu scattering pathways. This observation is in contradiction with the WT EXAFS obtained for the Cu(1.2)MOR(46) sample, which shows a significant decrease of the intensity of the lobe at (11.0 Å^−1^; 2.6 Å), assigned to a copper scatterer, as compared to the copper-exchanged mordenites with a higher aluminum content. We therefore assume that the results of FT EXAFS fitting for Cu(1.2)MOR(46) might be partially compromised by the low signal-to-noise ratio of the corresponding spectrum in combination with the low intensity of the scatters, which is evidenced by the high *R*-factor, returned after the fitting (Table 3, Fig. S18†). For a conclusive discussion, an even higher quality of the spectra is needed, which is a challenging task due to the low copper content in the material. Qualitatively, we rely on the results of WT EXAFS that show a very small impact of copper scattering at high *k*-values, suggesting the formation of copper monomeric species, in line with our previous studies devoted to infrared spectroscopy of adsorbed nitrogen monoxide, which is considered as the probe, sensitive to the nuclearity of copper species in zeolites.^15,56,58^ We showed that the spectra of adsorbed NO are very similar for Cu(4.4)MOR(6) and Cu(3.4)MOR(10), revealing the presence of identical bands with similar intensities, while Cu(1.2)MOR(46) gives a dramatically different spectrum. In the region of the Cu^II^ species, only one intense band at 1908 cm^−1^ was observed, hence indicating the dominant formation of monomeric copper species, whereas for samples with Si/Al ratios greater than 10, copper sites of different nuclearity were present.^15^

**Table 3:** Best-fit parameters optimized by EXAFS fits of the *k*^3^-weighted spectrum of activated copper-exchanged mordenite with different Si/Al ratios. DFT-optimized geometry for dicopper mono-μ-oxo sites located in the 8-membered ring of MOR was used as the starting guess. The fit was performed in *R*-space in the range of 1.0–3.0 Å, employing the *k*-range of 3.0–16.0 Å^−1^ for the FT, resulting in a number of independent points *N*_ind_ > 16

Parameter	Scatter	Cu(4.3)MOR(6)	Cu(3.4)MOR(10)	Cu(1.2)MOR(46)
Δ*E*, eV		+1.7(6)	+2.9(7)	−2.8(13)
*N*_O_	O	3.9(2)	3.7(2)	3.9(4)
*R*(Cu–O), Å		1.938(3)	1.944(3)	1.917(7)
Debye–Waller factor, 10^−3^ Å^2^		6.8(4)	6.3(3)	9.2(9)
*N*_Al_	Al or Si	1.8(7)	1.7(5)	0.5(3)
*R*(Cu–Al, Si), Å		2.69(2)	2.70(1)	2.64(2)
Debye–Waller factor, 10^−3^ Å^2^		11.3(60)	10.8(38)	4.1(37)
*N*_Cu_	Cu	0.3(4)	0.25(7)	0.3(3)
*R*(Cu–Cu), Å		2.86(3)	2.84(2)	2.87(2)
Debye–Waller factor, 10^−3^ Å^2^		7.1(68)	5.0(21)	5.0(49)
*R*-factor		0.0022	0.0029	0.0072
*χ* ^2^-parameter		111	187	207

Finally, the effect of copper loading on the FT EXAFS fits and, hence, the structure of copper-oxo species, was evaluated using a series of copper-exchanged mordenite samples of Si/Al = 6 with different copper loadings (Table 4, Fig. S19–S22†). The fitted Cu–O, Cu–Al and Cu–Cu distances over all samples give the values of 1.94, 2.66 and 2.85 Å, respectively. The first shell coordination number for copper surrounded by oxygen slightly decreases from 3.9 to 3.7 with decreasing copper content, which falls within the relative accuracy of EXAFS. Similarly, the second shell aluminum/silicon coordination number changes from 1.8 to 1.2. Notably, the copper–copper coordination number is preserved and does not vary from Cu(4.3)MOR(6) to Cu(1.7)MOR(6). These data show that the ion-exchange of mordenite with different copper concentrations results in the observation of qualitatively and quantitatively identical EXAFS spectra. In agreement with that, the Cu K-edge XANES spectra (Fig. 1c) demonstrate a similar intensity of pre-edge peaks and structure of the white line. Combining these data with recent results of infrared spectroscopy of adsorbed nitrogen monoxide, we conclude that the structure of copper-oxo species in this series of the samples is very similar and EXAFS and IR NO are not sensitive enough to detect any possible difference.

**Table 4:** Best-fit parameters optimized by EXAFS fits of the *k*^3^-weighted spectrum of activated copper-exchanged mordenite with different copper loadings. DFT-optimized geometry for dicopper mono-μ-oxo sites located in the 8-membered ring of MOR was used as the starting guess. The fit was performed in *R*-space in the range of 1.0–3.0 Å, employing the *k*-range of 3.0–16.0 Å^−1^ for the FT, resulting in a number of independent points *N*_ind_ > 16

Parameter	Scatter	Cu(4.3)MOR(6)	Cu(3.5)MOR(6)	Cu(3.2)MOR(6)	Cu(2.5)MOR(6)	Cu(1.7)MOR(6)
Δ*E*, eV		+1.7(6)	+2.1(6)	+2.6(7)	+1.4(12)	+1.5(12)
*N*_O_	O	3.9(2)	3.8(1)	3.6(2)	3.6(3)	3.7(3)
*R*(Cu–O), Å		1.938(3)	1.940(3)	1.944(3)	1.933(1)	1.93(2)
Debye–Waller factor, 10^−3^ Å^2^		6.8(4)	6.1(3)	6.0(4)	6.4(6)	6.1(6)
*N*_Al_	Al or Si	1.8(7)	1.6(3)	1.5(3)	1.1(6)	1.2(7)
*R*(Cu–Al, Si), Å		2.69(2)	2.66(1)	2.67(1)	2.66(2)	2.66(2)
Debye–Waller factor, 10^−3^ Å^2^		11.3(60)	8.3(21)	7.5(20)	8.0(49)	8.6(58)
*N*_Cu_	Cu	0.3(4)	0.2(2)	0.1(1)	0.4(5)	0.3(4)
*R*(Cu–Cu), Å		2.86(3)	2.86(1)	2.87(1)	2.88(3)	2.85(2)
Debye–Waller factor, 10^−3^ Å^2^		7.1(68)	4.6(36)	2.7(33)	7.6(77)	5.1(20)
*R*-factor		0.0022	0.0021	0.0031	0.0089	0.0098
*χ* ^2^-parameter		111	95	73	64	83

## 5. Conclusions

We have shown that Cu K-edge X-ray absorption spectroscopy in both XANES and EXAFS applications is a sensitive tool, enabling to discriminate different types of copper-oxo species in copper-exchanged zeolites. Collection of EXAFS data in the long *k*-range and at cryogenic temperatures significantly increases the quality and accuracy of the fitting, allowing quantitative interpretation of the spectra. Moreover, advanced post-processing of EXAFS data with wavelet transform constitutes a major step forward by observation of different scatterers, which contributes to a coordination sphere and, hence, facilitates the selection of appropriate models for conventional Fourier transform fitting.

Applying our approach, we observed a strong dependence of copper speciation on the zeolite topology. We established that zeolites BEA and FAU form copper-oxo clusters upon ion exchange and high-temperature oxidative treatment. In contrast, CuMOR revealed the presence of mostly copper monomeric species, while CuMFI demonstrated the formation of a significant fraction of copper-oxo dimers. A moderate variation of the Si/Al ratio or copper loading in copper-exchanged mordenite did not reveal a conclusive difference in either EXAFS or XANES spectra. A drastic increase of the Si/Al ratio in mordenite results in a very low copper loading in the final material, making the collection of a high quality spectrum and its unambitious analysis challenging. Our study identifies limitations and opportunities in the application of XAS by showing the great potential of wavelet analysis of EXAFS spectra of copper-containing materials and offers a methodology for the structural analysis of copper species stabilized in zeolites.

## Conflicts of interest

There are no conflicts to declare.

## Supplementary Material

SC-011-D0SC01472A-s001
