# Supplementary material for: Structure of copper sites in zeolites examined by Fourier and wavelet transform analysis of EXAFS
Source: Chem Sci. 2020 May 1;11(20):5299–312. doi: 10.1039/d0sc01472a (PMC8159279; doi:10.1039/d0sc01472a)
Supplement: SC-011-D0SC01472A-s001 [file SC-011-D0SC01472A-s001.pdf]

Electronic Supplementary Material (ESI) for Chemical Science.
This journal is © The Royal Society of Chemistry 2020

Supporting Information for

Structure of Copper sites in Zeolites Examined by Fourier and Wavelet Transform Analysis of EXAFS

Vitaly L. Sushkevich[a,*], Olga V. Safonova[b], Dennis Palagin[a], Mark A. Newton[c], Jeroen A. van Bokhoven[a,c,*]

[a] *Laboratory for Catalysis and Sustainable Chemistry, Paul Scherrer Institut, 5232 Villigen PSI, Switzerland*

[b] *Laboratory for Operando Spectroscopy, Paul Scherrer Institut, 5232 Villigen PSI, Switzerland*

[c] *Institute for Chemistry and Bioengineering, ETH Zurich, Vladimir-Prelog-Weg 1, 8093 Zurich, Switzerland*

Computational Details

All ground-state total energy calculations in this work have been performed with the all-electron full-potential DFT code FHI-aims (*S1, S2*) within the periodic boundary conditions model. Electronic exchange and correlation was treated on the hybrid functional level with the PBE0 functional (*S3*). All geometry optimization were done with the "tier2" atom-centered basis set using "tight" settings for numerical integrations. Tkatchenko-Scheffler dispersion correction (*S4*) has been used to account for the van der Waals energies arising from the attraction between induced dipoles formed due to charge fluctuations in the interacting species. Mordenite geometries reported herein correspond to the locally optimized configurations. Faujasite geometries correspond to locally optimized configurations under the assumption that the copper oxide cluster inside of the pore exhibits the C_{2v} symmetry, consistent with the experimentally observed bond lengths distribution.

Mordenite centers correspond to the periodic model of the mordenite structure, having 8- and 12-ring channels running parallel to the *c* axis, which are intersected by sinusoidal 8-ring channels that run parallel to the *b* axis. These form side pockets that connect the 12-ring channel with the 8-ring channel. There are two 12-ring and two 8-ring channels per unit cell of mordenite. Two symmetrically located aluminium atoms per 8-ring channel have been assumed, which corresponds to the Si/Al ratio of 11.

Faujasite centers correspond to the periodic model, composed of sodalite cages with diameter of 6.6 Å connected to supercages having a diameter of 12.4 Å. These two units are interconnected by hexagonal prisms whose opening is of 2.3 Å. These supercages are linked together by a 12MR ring with diameter of 7.4 Å, forming the porous accessible network.

Wavelet transform details

For the continuous wavelet transform of EXAFS data Morlet mother function was used:

$$\psi(x) = 1/\sqrt[4]{\pi} cos(x\pi\sqrt{2/log(2)}) exp(-\frac{x^2}{2}),$$

which corresponds to the frequency ω = $\pi\sqrt{2/log(2)}$ and bandwidth $\sigma^2 = 1$. The used frequency in close to the frequencies of backscattered waves making the resolution in k-space of WT better.

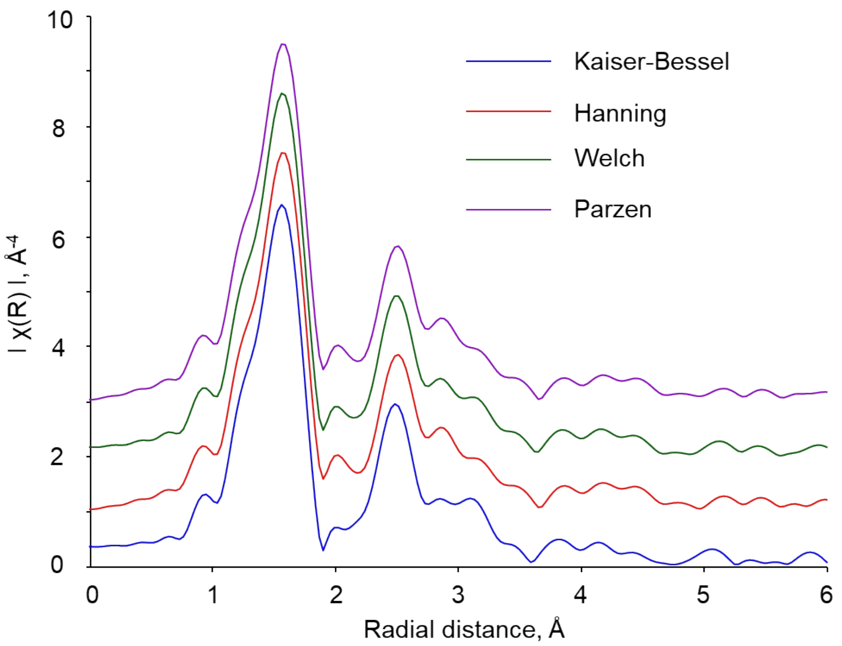

Fig. S1. Results of the Fourier transform of the k^3-weighted EXAFS spectrum of Cu(2.7)FAU(15) with different apodization window functions. It shows the splitting observed for the second coordination sphere is not a function of applied apodization.

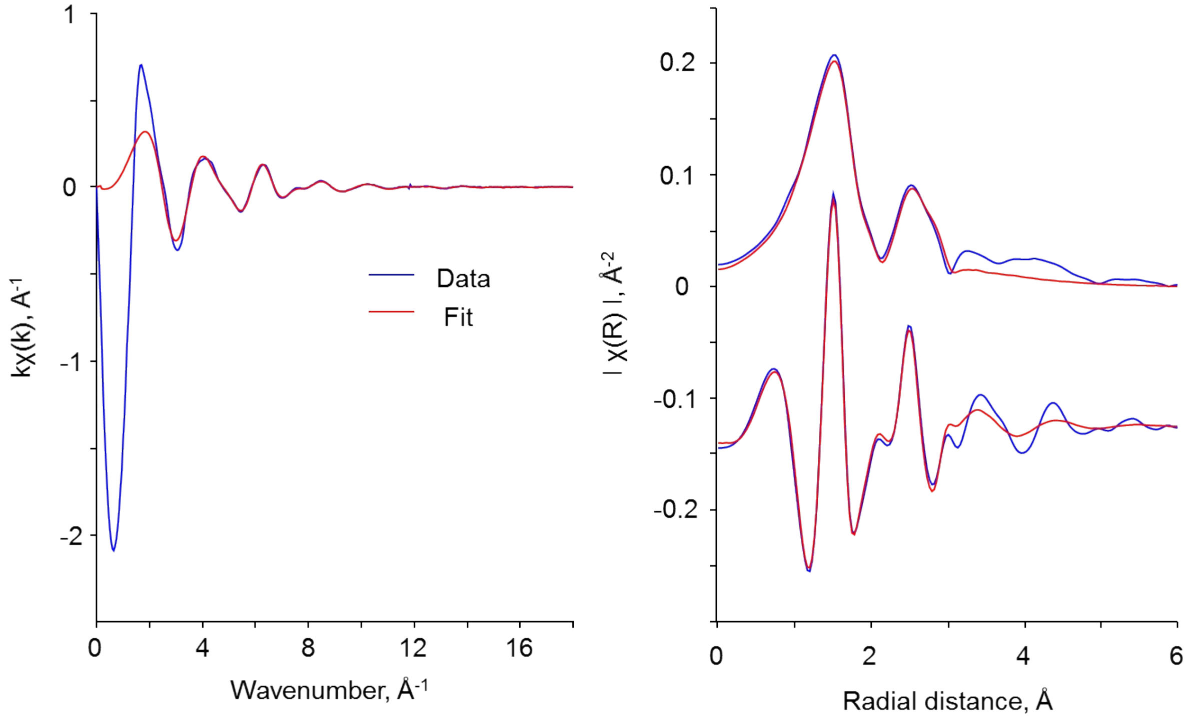

Fig. S2. Results of the fitting of k^3-weighted FT EXAFS spectrum of Cu(3.4)MOR(10), displayed with k^1-weightening. Left part corresponds to the fitted k-space and right part shows R-space magnitudes and real part of FT. The fit was performed in R-space, in the range of 1.0–3.0 Å, employing the k-range of 3.0–16.0 $Å^{-1}$ for the FT. The absence of additional peaks in k^1 data indicated the negligible contribution of multiple scattering pathways to the EXAFS spectrum.

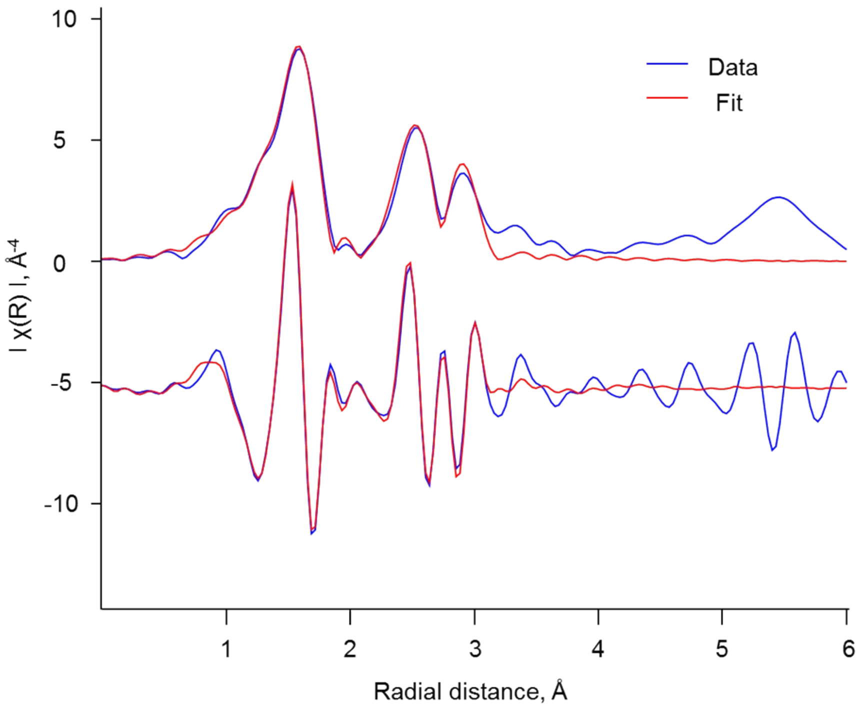

Fig. S3. Results of the fitting of k^3-weighted FT EXAFS spectrum of CuO standard. The fit was performed in R-space, in the range of 1.0–3.3 Å, employing the k-range of 3.0–16.4 $Å^{-1}$ for the FT. Five main single scattering paths extracted from tenorite crystallographic structure were used for the fitting. The details of the fit are given in Table S1. The obtained data points to the negligible contribution of multiple scattering paths in the range of 1.0–3.3 Å.

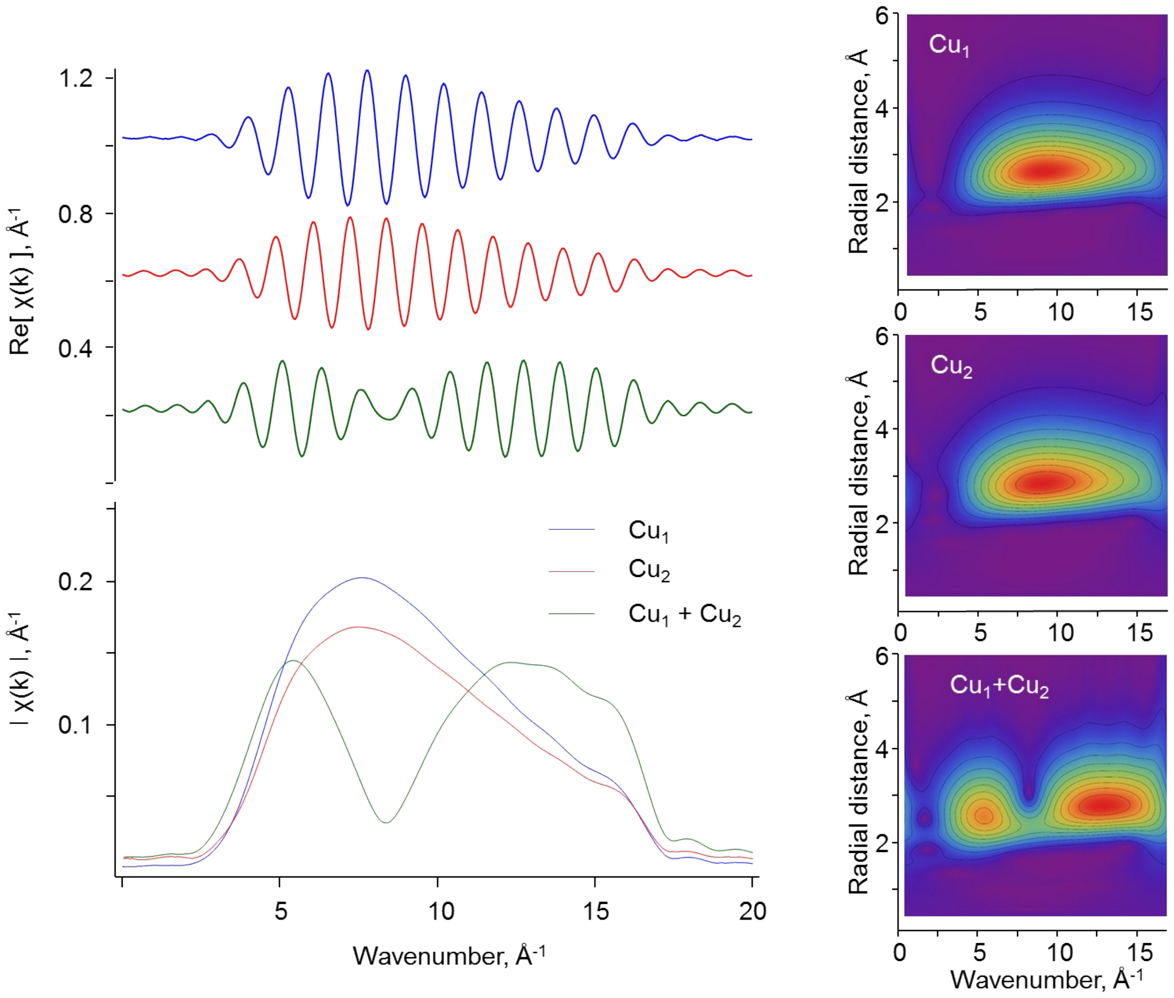

Fig. S4. Real parts of backscattering waves, their wavelet transforms and corresponding amplitudes obtained by simulation of scattering paths from copper atoms in copper (II) oxide, remoted by 2.88 and 3.07 A from the absorber.

For the atoms of a particular nature, having a low to moderate Z number, there is a single main maximum on the curve describing backscattering factor dependence on k value. This is indeed the case for the isolated atoms. However, in case, multiple non-equivalent atoms of similar nature are located at similar distances, the addition of backscattered waves can lead to destructive interference, which results in the appearance of several maxima on the corresponding magnitude curve.

As an example, the magnitude of the wave, obtained by a combination of two backscattering waves from copper atoms in copper oxide is shown. It is clear, that those two atoms individually have magnitudes with one maximum. However, the sum of these waves results in beating and the appearance of two maxima on the magnitude curve and, respectively, in the WT counterplots.

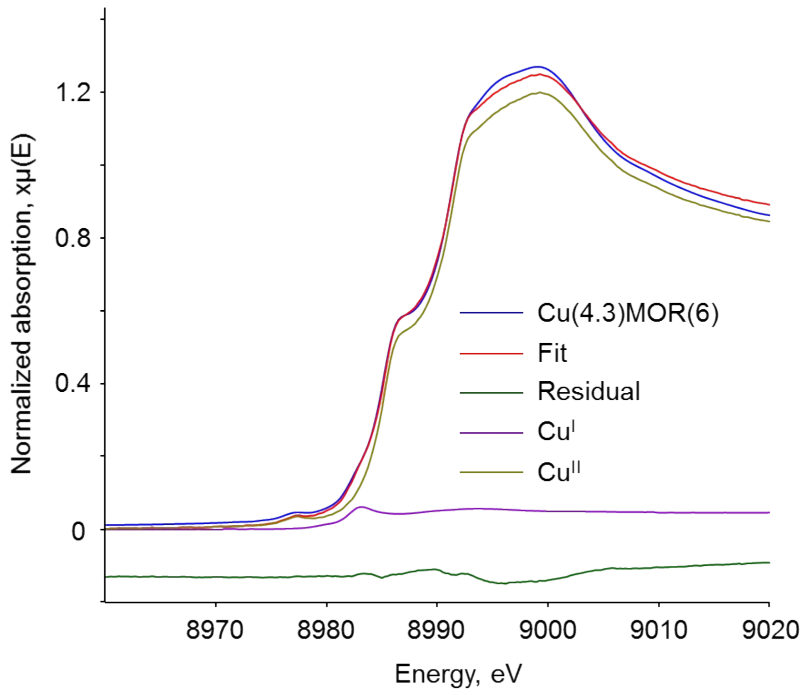

Fig. S5. Results of linear combination fitting analysis of XANES spectrum for the Cu(4.3)MOR(6), using two standards obtained by the treatment: i) in oxygen at 773K and ii) in methane at 773K for 1h. It returns the Cu^{I} concentration of 3.4%.

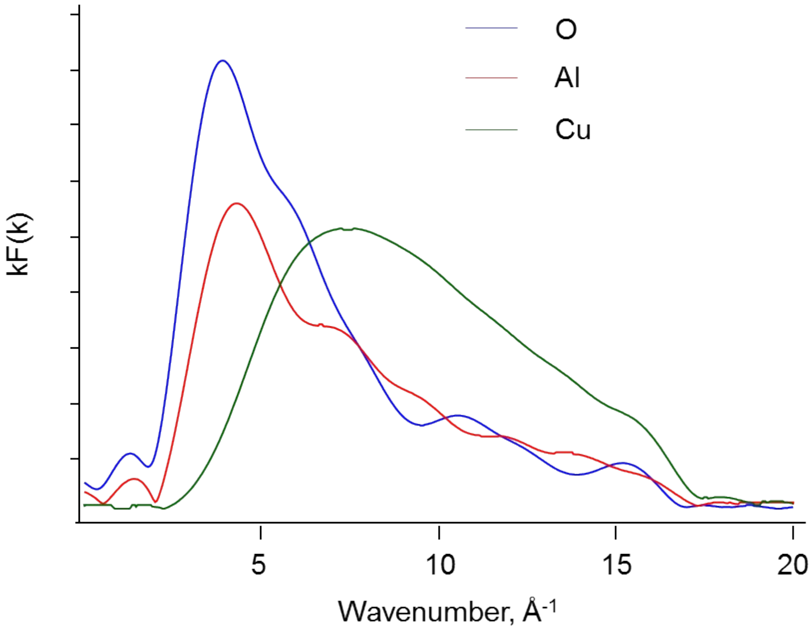

Fig. S6. Plot of the k-weighted backscattering amplitude factor calculated for single scattering paths with copper atom as absorber. For the calculations, dicopper mono-μ-oxo species located in an 8-membered ring of mordenite, optimized with density functional theory (DFT) was used.

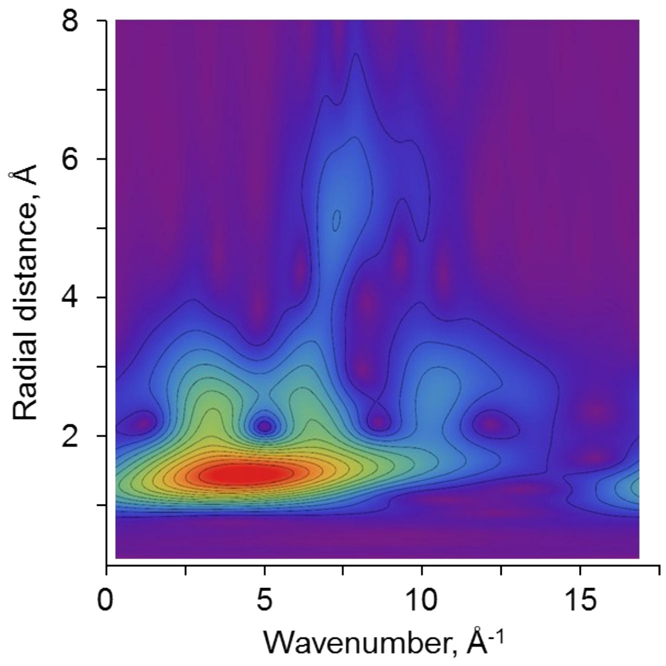

Fig. S7. 2D plots of WT EXAFS for k^2-weighted $\chi(k)$ data for CuO.

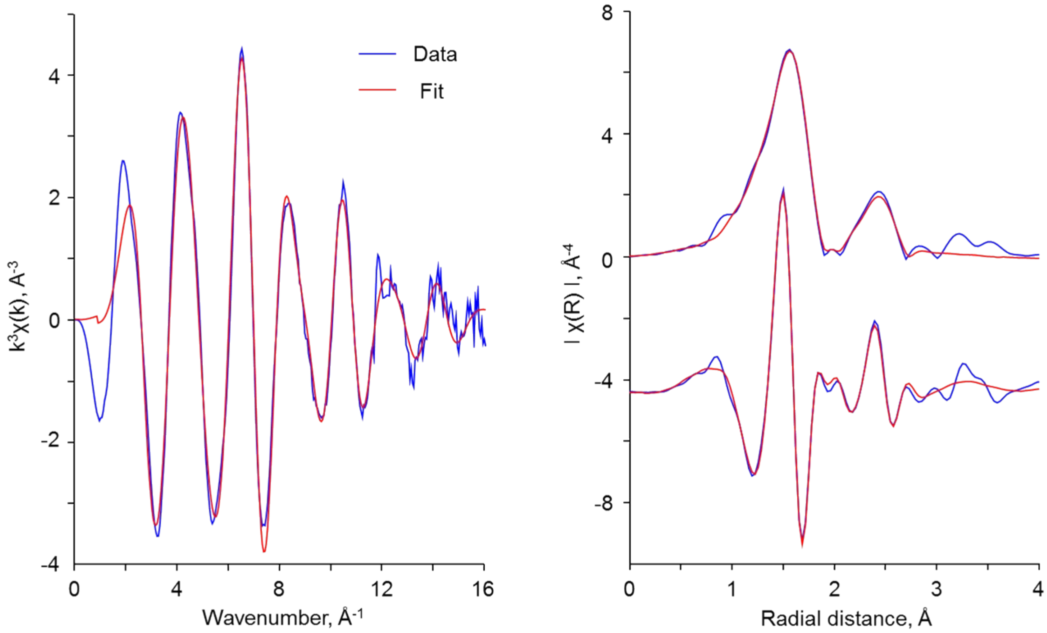

Fig. S8. Results of the fitting of k^3-weighted FT EXAFS spectrum of Cu(3.4)MOR(10). Left part corresponds to the fitted k-space and right part shows R-space magnitudes and real part of FT. The fit was performed in R-space, in the range of 1.0–3.0 Å, employing the k-range of 3.0–16.0 $Å^{-1}$ for the FT.

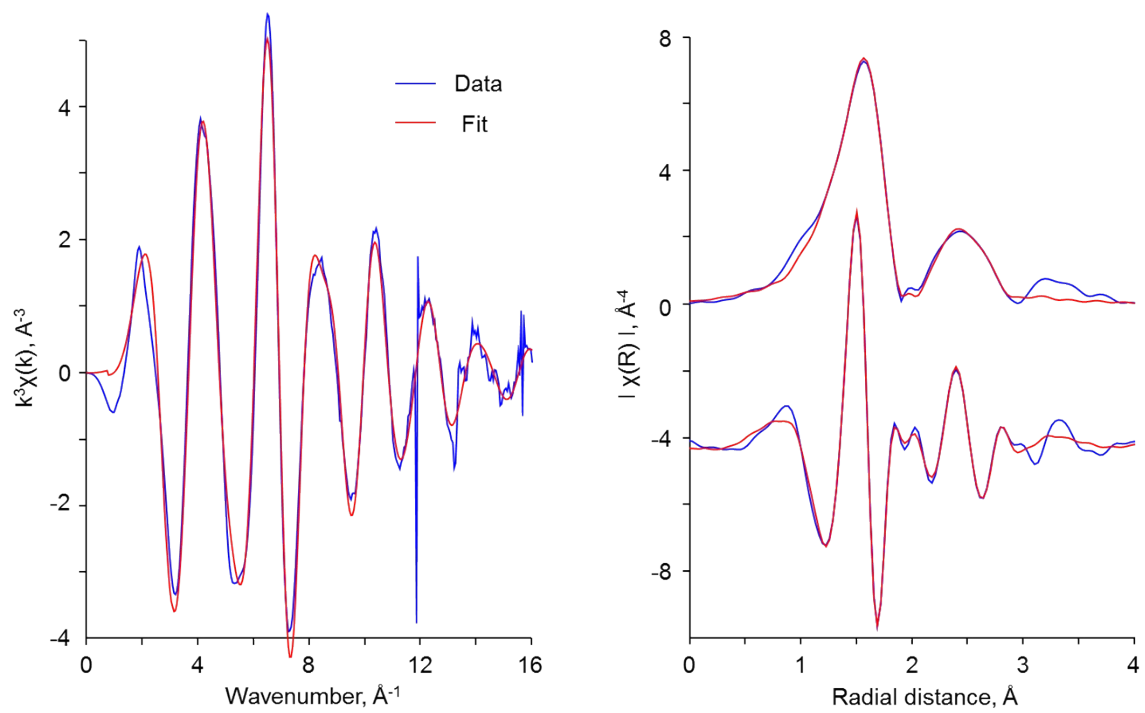

Fig. S9. Results of the fitting of k^3-weighted FT EXAFS spectrum of Cu(4.0)MFI(12). Left part corresponds to the fitted k-space and right part shows R-space magnitudes and real part of FT. The fit was performed in R-space, in the range of 1.0–3.0 Å, employing the k-range of 3.0–16.0 $Å^{-1}$ for the FT.

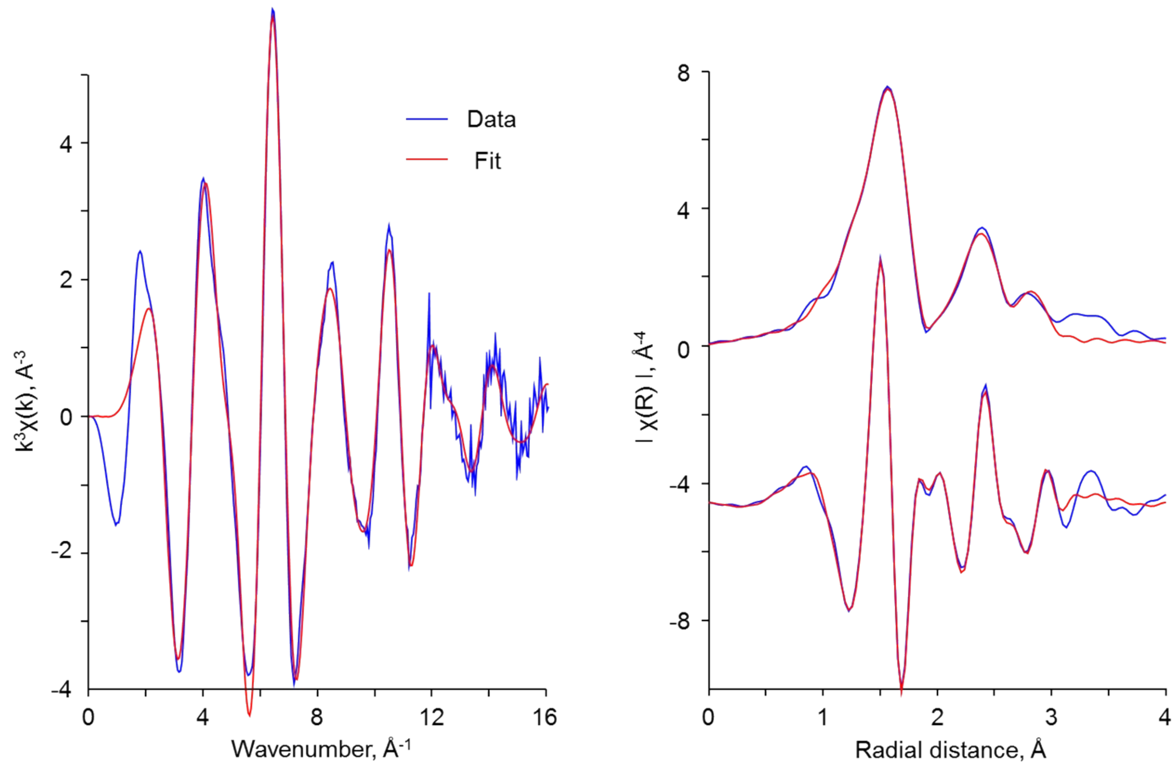

Fig. S10. Results of the fitting of k^3-weighted FT EXAFS spectrum of Cu(2.8)BEA(12). Left part corresponds to the fitted k-space and right part shows R-space magnitudes and real part of FT. The fit was performed in R-space, in the range of 1.0–3.2 Å, employing the k-range of 3.0–16.0 $Å^{-1}$ for the FT.

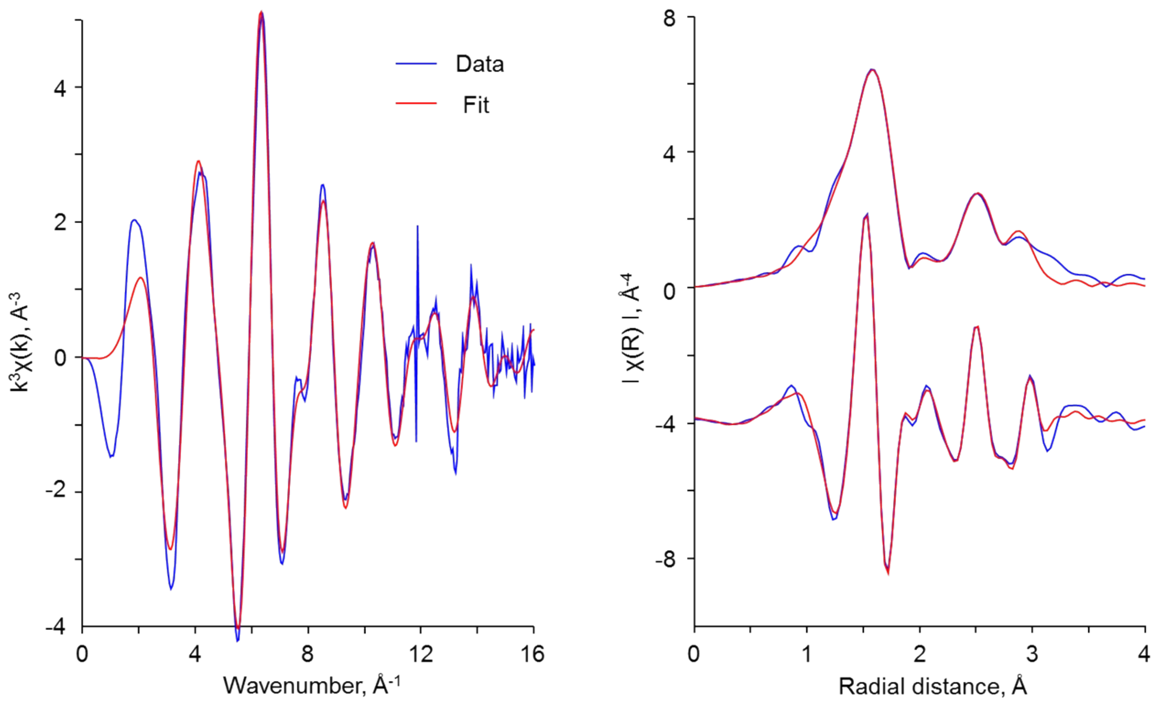

Fig. S11. Results of the fitting of k^3-weighted FT EXAFS spectrum of Cu(2.7)FAU(15). Left part corresponds to the fitted k-space and right part shows R-space magnitudes and real part of FT. The fit was performed in R-space, in the range of 1.0–3.2 Å, employing the k-range of 3.0–16.0 $Å^{-1}$ for the FT.

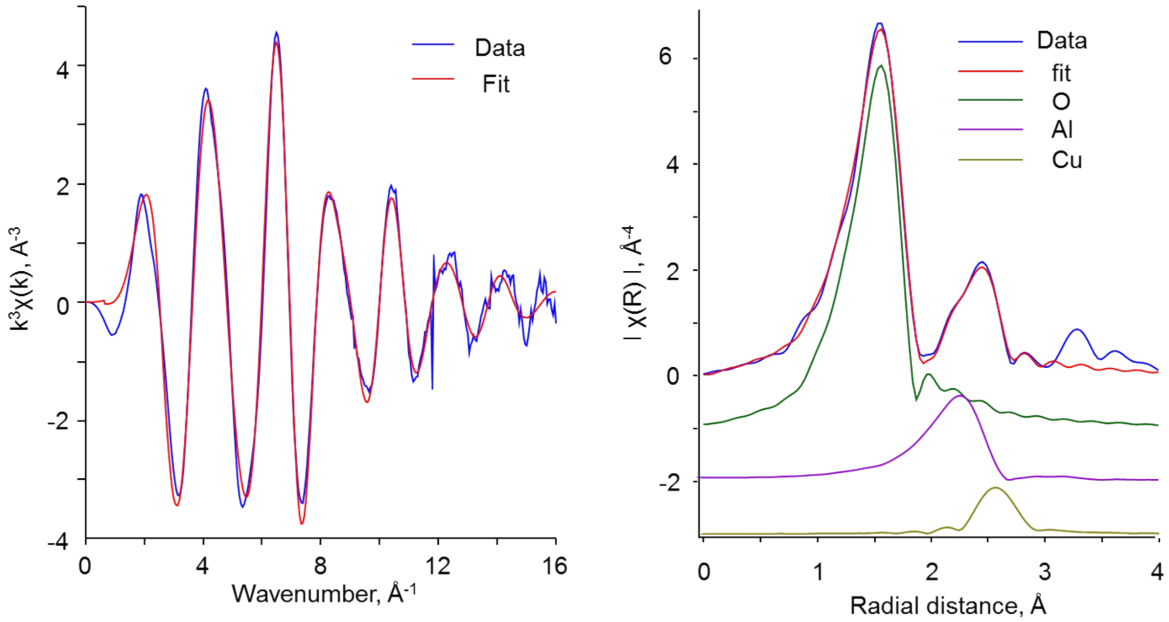

Fig. S12. Results of the fitting of k^3-weighted FT EXAFS spectrum of Cu(3.4)MOR(10) using both Al and Cu scatters contributing to the second coordination shell. Left part displays the k-space data and the resulting fit, while right part shows the magnitudes due to oxygen, aluminum and copper atoms, impacting to the resulting FT EXAFS.

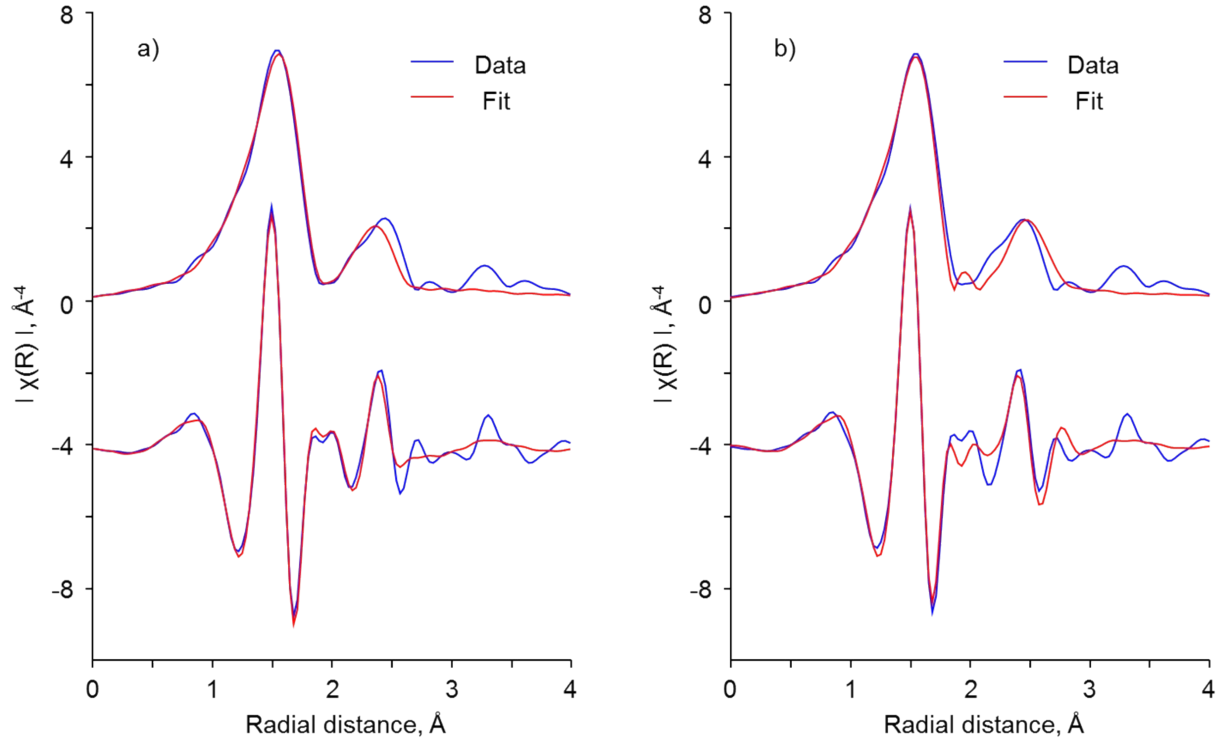

Fig. S13. Examples of k^3-weighted EXAFS fits of Cu(4.3)MOR(6) material: a) with exclusive contribution of Al in the second shell and b) with exclusive contribution of Cu. A clear misfits are visible, which can only be avoided by the fitting of the second shell with simultaneous contribution from both Al and Cu.

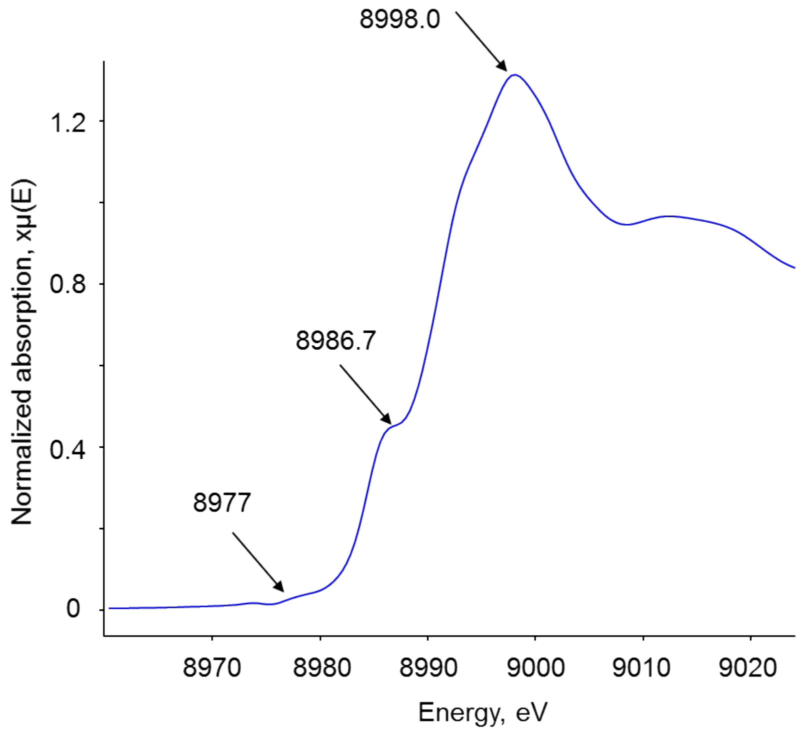

Fig. S14. Cu K-edge XANES spectrum of bulk CuO, measured at 298 K.

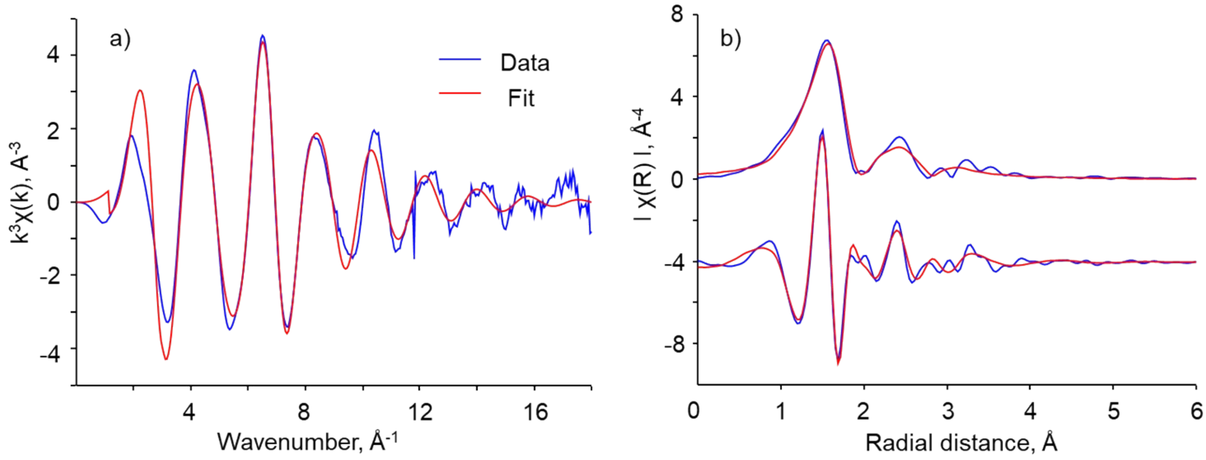

Fig. 15. Results of the fitting of k^3-weighted FT EXAFS spectrum of Cu(4.3)MOR(6) using the fit obtained for Cu(2.7)FAU(15) as the initial guess with fixed interatomic distances. Left part corresponds to the fitted k-space and right part shows R-space magnitudes and real part of FT.

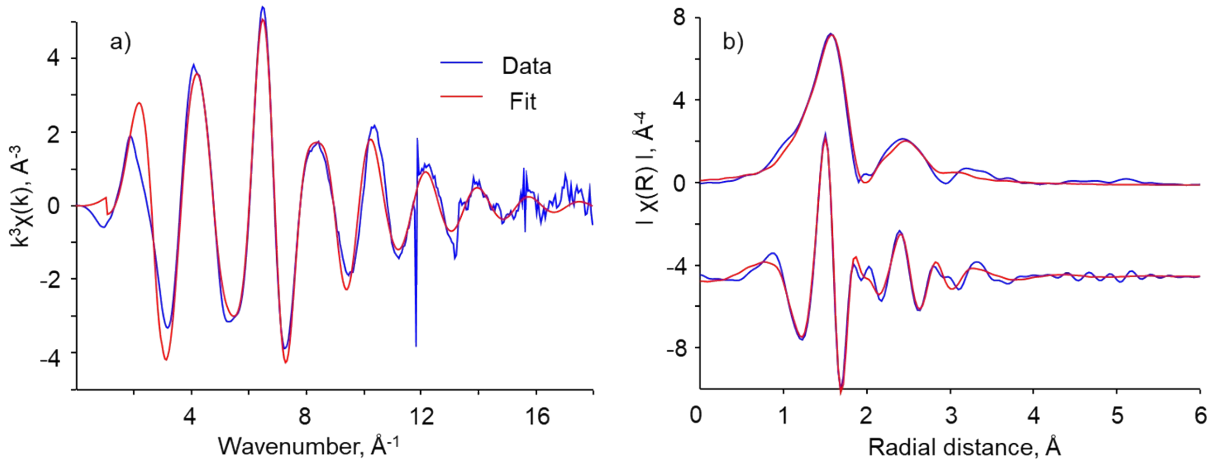

Fig. S16. Results of the fitting of k^3-weighted FT EXAFS spectrum of Cu(4.0)MFI(12) using the fit obtained for Cu(2.7)FAU(15) as the initial guess with fixed interatomic distances. Left part corresponds to the fitted k-space and right part shows R-space magnitudes and real part of FT.

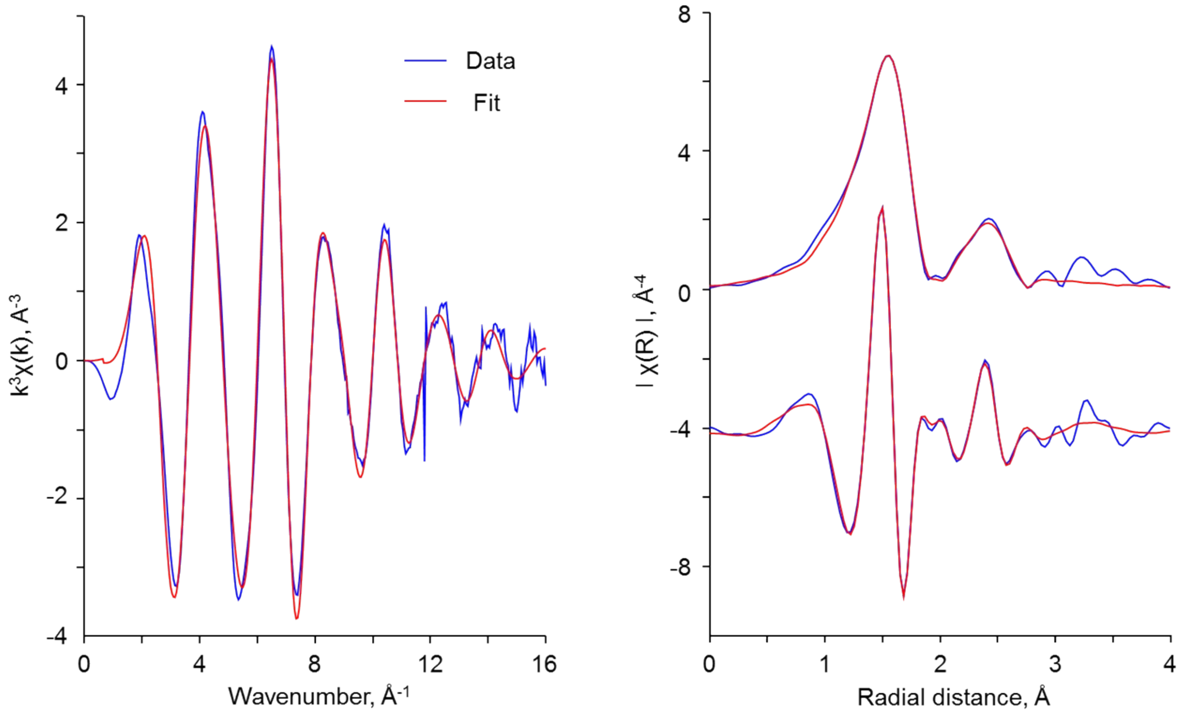

Fig. S17. Results of the fitting of k^3-weighted FT EXAFS spectrum of Cu(4.3)MOR(6). Left part corresponds to the fitted k-space and right part shows R-space magnitudes and real part of FT. The fit was performed in R-space, in the range of 1.0–3.0 Å, employing the k-range of 3.0–16.0 $Å^{-1}$ for the FT.

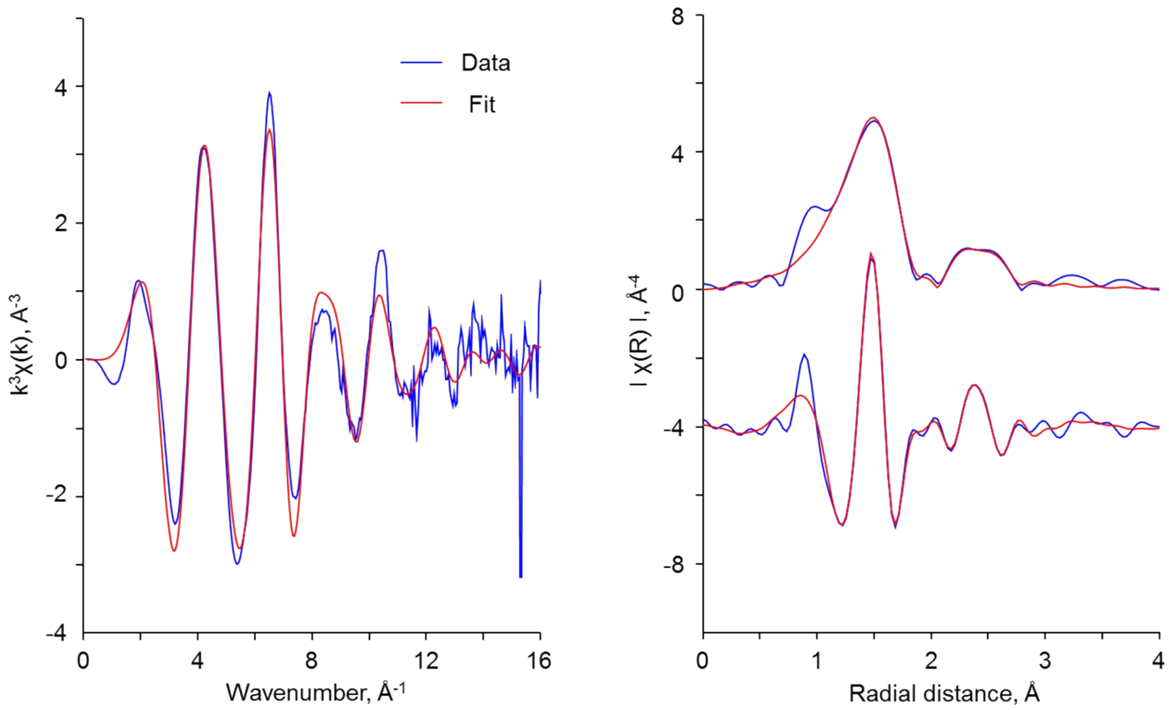

Fig. S18. Results of the fitting of k^3-weighted FT EXAFS spectrum of Cu(1.2)MOR(46). Left part corresponds to the fitted k-space and right part shows R-space magnitudes and real part of FT. The fit was performed in R-space, in the range of 1.0–3.0 Å, employing the k-range of 3.0–16.0 $Å^{-1}$ for the FT.

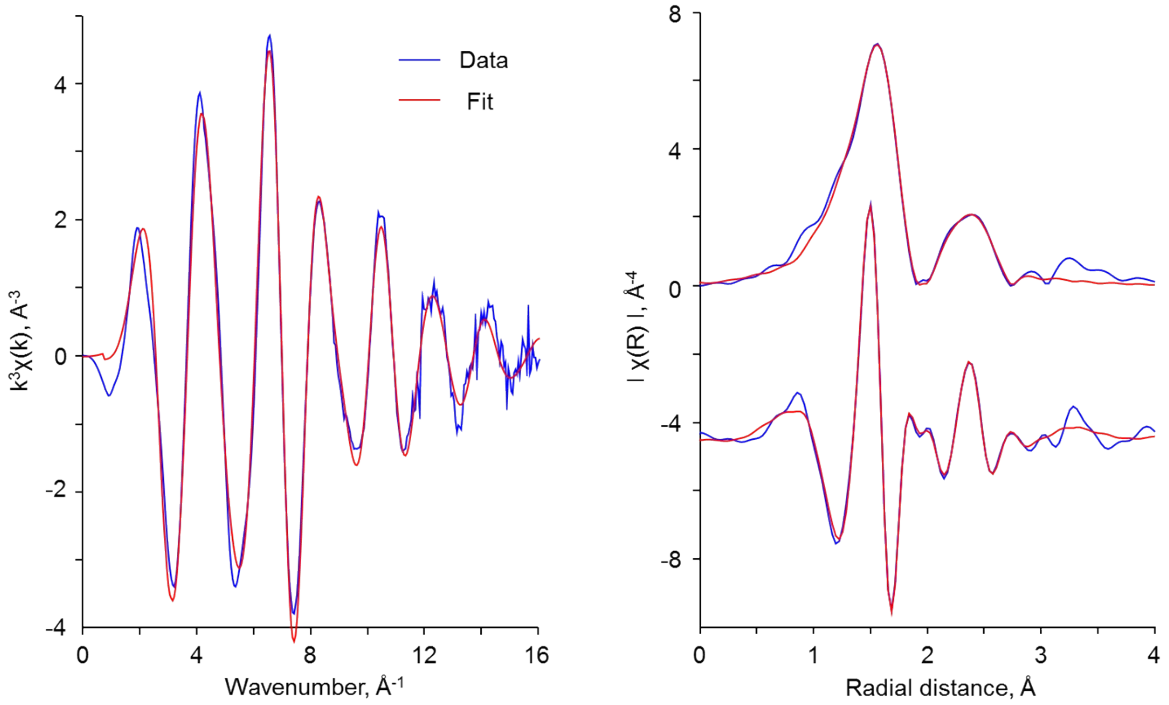

Fig. S19. Results of the fitting of k^3-weighted FT EXAFS spectrum of Cu(3.5)MOR(6). Left part corresponds to the fitted k-space and right part shows R-space magnitudes and real part of FT. The fit was performed in R-space, in the range of 1.0–3.0 Å, employing the k-range of 3.0–16.0 $Å^{-1}$ for the FT.

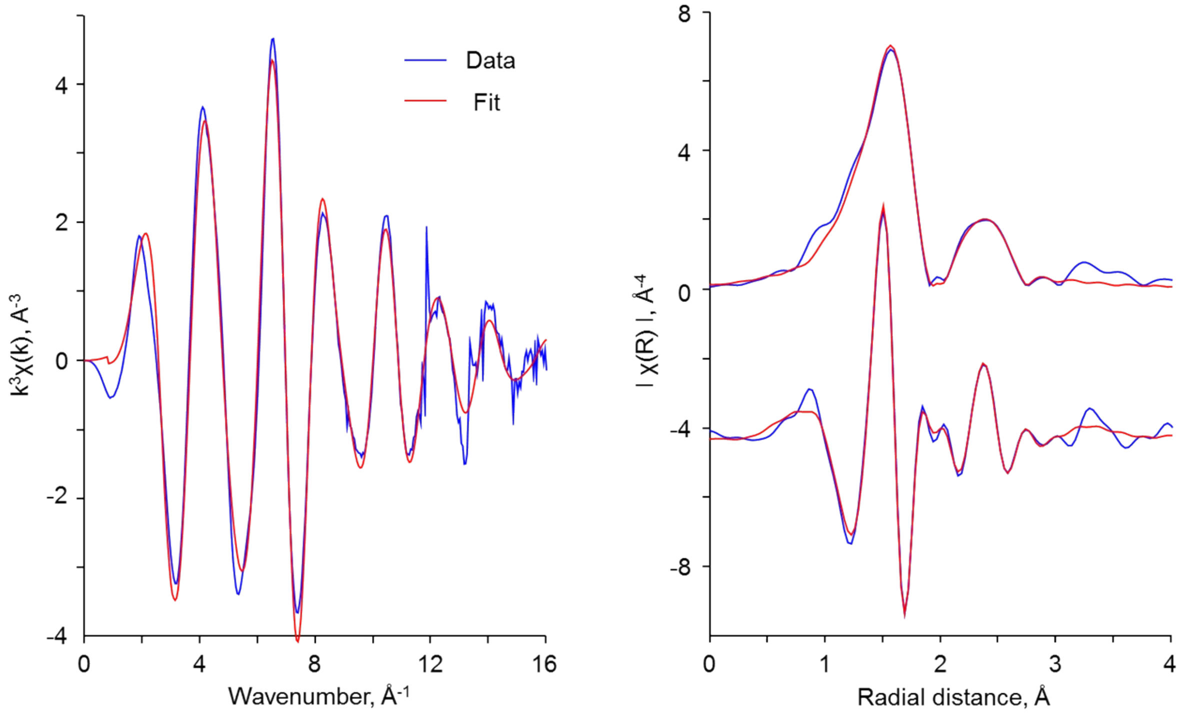

Fig. S20. Results of the fitting of k^3-weighted FT EXAFS spectrum of Cu(3.2)MOR(6). Left part corresponds to the fitted k-space and right part shows R-space magnitudes and real part of FT. The fit was performed in R-space, in the range of 1.0–3.0 Å, employing the k-range of 3.0–16.0 $Å^{-1}$ for the FT.

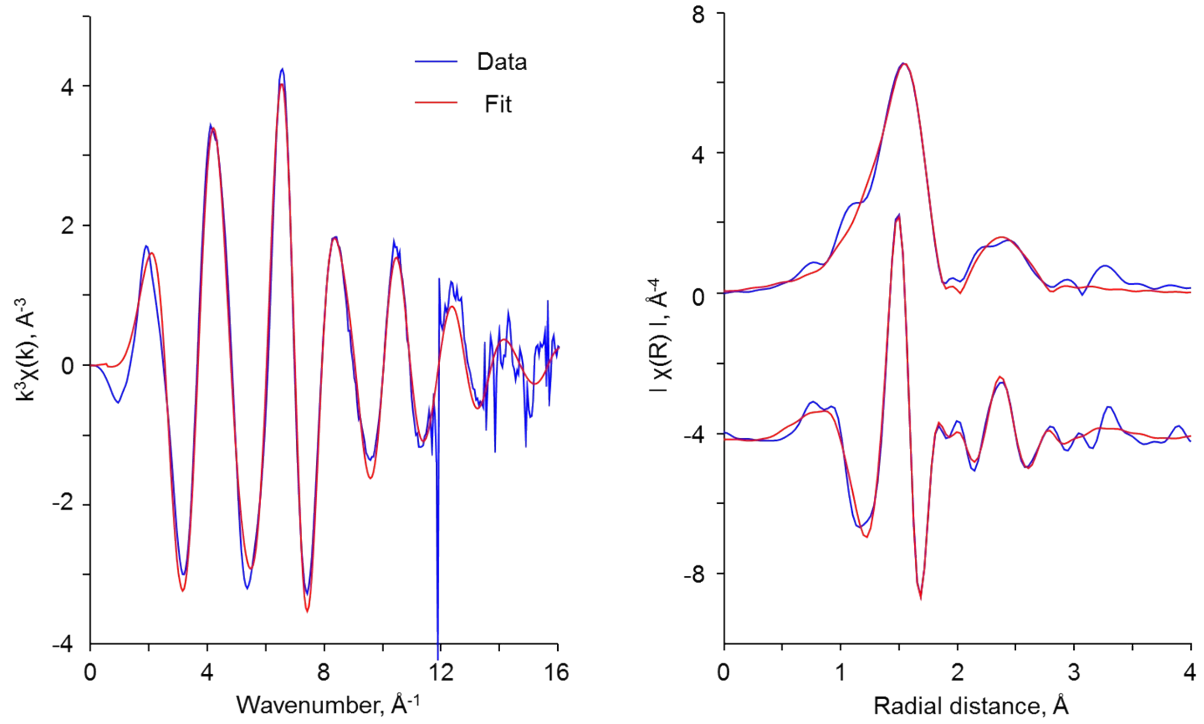

Fig. S21. Results of the fitting of k^3-weighted FT EXAFS spectrum of Cu(2.5)MOR(6). Left part corresponds to the fitted k-space and right part shows R-space magnitudes and real part of FT. The fit was performed in R-space, in the range of 1.0–3.0 Å, employing the k-range of 3.0–16.0 $Å^{-1}$ for the FT.

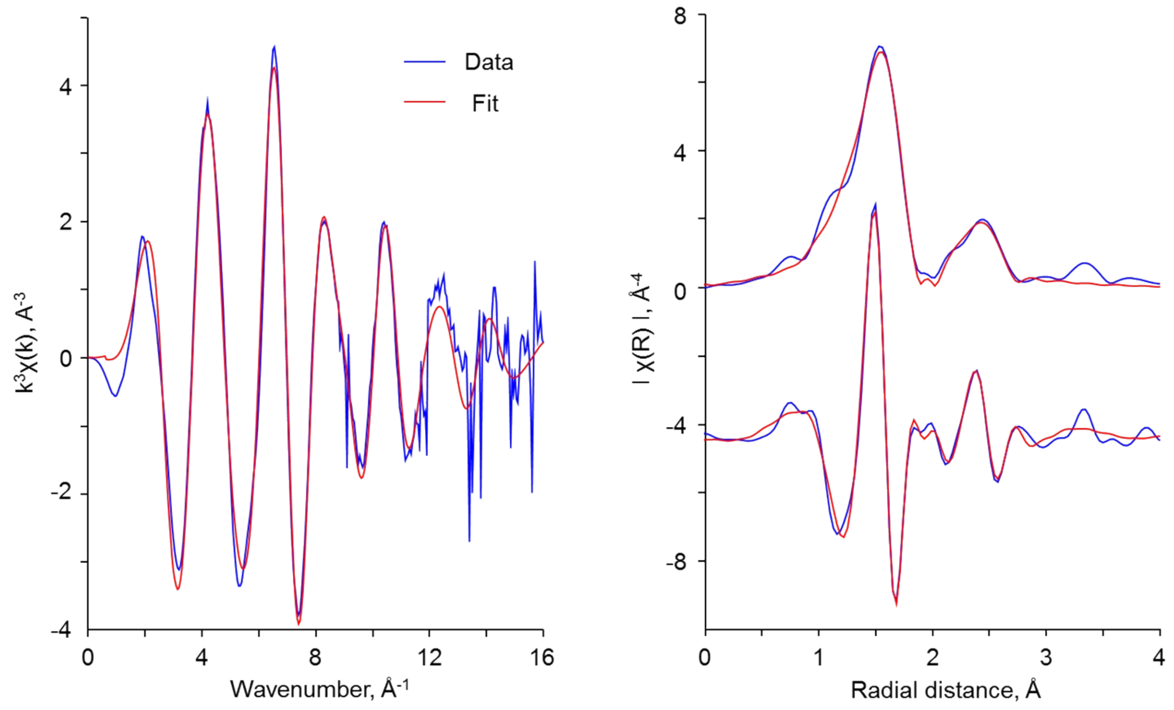

Fig. S22. Results of the fitting of k^3-weighted FT EXAFS spectrum of Cu(1.7)MOR(6). Left part corresponds to the fitted k-space and right part shows R-space magnitudes and real part of FT. The fit was performed in R-space, in the range of 1.0–3.0 Å, employing the k-range of 3.0–16.0 $Å^{-1}$ for the FT.

Table S1. Best-fit parameters optimized by EXAFS fits of the k^3-weighted spectrum of standard CuO. The tenorite crystallographic structure was used to generate scattering paths. Degeneracies were fixed according to the crystal structure. Other parameters, including Debye-Waller factors and effective radii were released. The fit was performed in R-space in the range of 1.0–3.3 Å, employing the k-range of 3.0–16.4 $Å^{-1}$ for the FT, resulting in a number of independent points $N_{ind} > 19$.

Parameter	Scatter	CuO
ΔE, eV		+0.1(9)
N_O		4
R(Cu-O_1), Å	O_1	1.953(5)
Debye-Waller factor, 10^{-3} $Å^2$		4(0)
N_{Al}		2
R(Cu-O_2), Å	O_2	2.74(6)
Debye-Waller factor, 10^{-3} $Å^2$		14(9)
N_{Cu}		4
R(Cu-Cu_1), Å	Cu_1	2.91(1)
Debye-Waller factor, 10^{-3} $Å^2$		8(1)
N_{Cu}		4
R(Cu-Cu_2), Å	Cu_2	3.06(5)
Debye-Waller factor, 10^{-3} $Å^2$		15(8)
N_{Cu}		2
R(Cu-Cu_2), Å	Cu_3	3.12(2)
Debye-Waller factor, 10^{-3} $Å^2$		6(2)
R-factor		0.012
χ^2-parameter		189

Table S2. Best-fit parameters optimized by EXAFS fits of the k^3-weighted spectrum of activated copper-exchanged MOR and MFI. The fit obtained for Cu(2.7)FAU(15) with fixed

atom coordinates was used as the starting guess. Other parameters, including Debye-Waller factors and amplitudes were released. The fit was performed in R-space in the range of 1.0–3.2 Å, employing the k-range of 3.0–16.0 $Å^{-1}$ for the FT, resulting in a number of independent points $N_{ind} > 16$.

Parameter	Scatter	Cu(4.3)MOR(6)	Cu(4.0)MFI(12)
ΔE, eV		+4.9(9)	+4.6(8)
N_O		4.2(5)	4.1(4)
R(Cu-O), Å	O	1.959(3)	1.959(4)
Debye-Waller factor, 10^{-3} $Å^2$		7.3(10)	6.5(1)
N_{Al}		12(7)	10(7)
R(Cu-Al, Si), Å	Al or Si	2.77	2.77
Debye-Waller factor, 10^{-3} $Å^2$		41(15)	40(16)
N_{Cu}		4.1(53)	4.3(26)
R(Cu-Cu_1), Å	Cu	2.93	2.93
Debye-Waller factor, 10^{-3} $Å^2$		28(14)	22(6)
N_{Cu}		-3.7(60)	-2.6(24)
R(Cu-Cu_2), Å	Cu	3.08	3.08
Debye-Waller factor, 10^{-3} $Å^2$		28(15)	22(6)
R-factor		0.025	0.0146
χ^2-parameter		1320	139

The obtained fits returned negative values for the second copper atom in the second coordination sphere, together with unrealistic coordination numbers for aluminum, indicating the absence of this scattering pathway in copper-exchanged MFI and MOR, hence confirming the different nature of copper-oxo species in zeolites of different structure.

Cartesian coordinates of the mono(μ-oxo)dicopper site in mordenite:

```
lattice_vector    18.25600000     0.00000000     0.00000000
lattice_vector     0.00000000    20.53400000     0.00000000
lattice_vector     0.00000000     0.00000000     7.54200000
atom     5.24642699    -0.10964989    -0.09536664 O
atom    13.95743362    10.39769246    -0.01163178 O
atom    12.96732457     0.18074116    -0.32314149 O
atom     4.18269061    10.43394489    -0.07714041 O
atom     4.95274291    -0.09710210     4.02627764 O
atom    14.53445099    10.65082711     3.89211269 O
atom    13.35787421     0.14517226     3.78595966 O
atom     3.88938607    10.09376579     3.52271479 O
atom     5.78268376     1.51043534     1.99546492 O
atom    15.54551403    11.81908162     1.66194991 O
atom    11.97503336     1.45019415     1.86468173 O
atom     3.19071922    12.08707331     1.84223244 O
atom     5.94224838    18.52355461     5.65607538 O
atom    15.04255323     8.71590677     5.67367657 O
atom    12.51996625    18.72679407     5.47054731 O
atom     2.80110678     8.94430633     5.73265246 O
atom     6.76156004     2.10495108     7.17330085 O
atom    15.86537859    11.96073413     6.55900319 O
atom    11.39048391     2.38879617     6.99527411 O
atom     2.65406467    12.62132954     6.87258865 O
atom     7.06269156    18.97920668     3.25248135 O
atom    16.24892350     8.62313814     3.31915091 O
atom    11.60623827    18.80579031     2.98617557 O
atom     1.75585323     8.38870176     3.38915436 O
atom    11.30291168    18.65453455     0.27851547 O
atom     2.21074500     8.83494529     0.77271425 O
atom     6.90188501    18.37455609     0.57342377 O
atom    15.76686976     8.52100273     0.64976918 O
atom    11.19770718     1.62319054     4.37874725 O
atom     2.15179006    12.00230169     4.27111193 O
atom     6.72866635     1.89212781     4.43060935 O
atom    16.56065041    12.47453251     4.01849150 O
atom     4.06402645     2.21943378     7.53319794 O
atom    13.67945385    13.06374351     7.75734893 O
atom    13.90705255     2.56374260     7.95874237 O
atom     5.18512261    12.85821457     7.77515447 O
atom     4.64575640    17.87744918     3.35473007 O
atom    13.57191315     8.21526949     3.45179172 O
atom    14.25279904    18.24489891     3.45437332 O
atom     4.22952603     7.51660640     3.91253904 O
atom    13.96719240    18.28462985     0.16888367 O
atom     4.55269274     7.78466567    -0.13296483 O
atom     4.23738297    18.02026881     0.13462424 O
atom    13.23322071     7.85241881    -0.08683814 O
```

atom	13.53116962	2.79697158	3.61076252	O
atom	4.80673581	12.53182791	3.96880927	O
atom	4.07195479	2.34732219	3.91704937	O
atom	14.04091841	13.20113806	3.38659297	O
atom	6.03104204	6.62579108	2.16802657	O
atom	14.97926584	16.32506981	1.69718168	O
atom	12.22099125	6.22624364	1.81494721	O
atom	3.27943013	16.16675136	1.82597638	O
atom	6.27912977	14.01211017	5.58102930	O
atom	15.03722980	3.48666452	5.72818693	O
atom	12.46791821	13.82547479	5.47843536	O
atom	3.18422183	4.07511659	5.78472598	O
atom	4.57187837	4.81913593	0.41977863	O
atom	13.53856150	15.68716900	-0.47112359	O
atom	13.91120505	5.23448600	-0.05433667	O
atom	4.59358496	15.40919389	-0.42666579	O
atom	4.56517018	15.22842710	3.94001924	O
atom	13.87736971	5.37303191	4.22821406	O
atom	13.45655988	15.76392042	3.88537879	O
atom	4.79646912	4.88709530	3.76872432	O
atom	7.01873249	6.20238639	7.38791269	O
atom	15.97511602	16.82375990	6.78371740	O
atom	11.40010726	6.00031839	6.88532583	O
atom	2.15510609	16.45534074	6.99585435	O
atom	6.80547306	13.98744440	2.97364861	O
atom	15.94475137	3.88592001	3.26581302	O
atom	11.55436266	14.09863285	2.99955920	O
atom	2.20448578	4.19001305	3.34877942	O
atom	11.33264120	14.32798713	0.29522435	O
atom	2.05390911	3.85108635	0.64155741	O
atom	7.07970147	14.76944442	0.39996244	O
atom	16.16376022	3.99132329	0.56183207	O
atom	11.25580717	6.53046221	4.20111990	O
atom	2.40930092	16.81132191	4.30156897	O
atom	6.56478406	6.62733644	4.83305790	O
atom	16.08619701	16.36298099	4.08327320	O
atom	-0.12622761	7.88101452	1.55328824	O
atom	9.22424139	18.52603880	1.84357364	O
atom	0.14945511	12.36472197	5.97925963	O
atom	9.01184103	2.05669017	5.81444546	O
atom	2.06081958	6.31301319	1.69002238	O
atom	11.01483592	16.47685254	1.85716149	O
atom	16.02737870	6.25584176	2.04271111	O
atom	7.51659656	16.49498928	2.41885262	O
atom	1.66393221	14.44494753	5.19857251	O
atom	10.60728805	4.11571743	5.10120008	O
atom	16.63999566	14.42376307	5.89291174	O
atom	7.36081126	4.18550029	5.58808942	O
atom	-0.04274931	4.64471135	2.10063515	O
atom	9.20945857	14.43386071	1.91164519	O

atom	0.02471339	16.57365908	5.48389108	O
atom	9.09148683	6.27066095	5.81511865	O
atom	5.45273324	1.44083081	0.38967001	Si
atom	14.75694312	11.82762900	0.21647521	Si
atom	12.55999577	1.62801375	0.33979752	Si
atom	3.79415289	11.97713130	0.31885749	Si
atom	5.64603073	18.94798483	4.09737914	Si
atom	14.82800172	9.03846371	4.07521696	Si
atom	12.92492723	19.09569772	3.92212537	Si
atom	3.17022409	8.75109604	4.15658160	Si
atom	12.69113448	19.09893181	7.05693987	Si
atom	3.46116218	8.96139033	7.24631602	Si
atom	5.59195858	18.84065391	7.23500831	Si
atom	14.50998201	8.87382381	7.20963315	Si
atom	12.53677767	1.52149542	3.41463805	Si
atom	3.51333864	11.69342037	3.39881209	Si
atom	5.38977203	1.42700102	3.58599576	Si
atom	15.16861924	12.02005035	3.24892129	Si
atom	5.30505009	6.37908166	0.50694633	Al
atom	14.61146312	16.76658478	0.15749108	Si
atom	12.74122457	6.32666977	0.22864880	Si
atom	3.58807492	16.51584515	0.25242189	Si
atom	5.60062128	13.96423929	4.08832588	Si
atom	14.58753792	3.91752299	4.20150355	Si
atom	12.89178459	14.21804555	3.94041196	Si
atom	3.56141063	3.88085179	4.20888773	Si
atom	12.77044639	14.22905902	7.04004086	Si
atom	3.49314637	3.76388054	7.37122441	Si
atom	5.76056652	14.25206815	7.11683366	Si
atom	14.74122648	3.81823337	7.30071205	Si
atom	12.94428858	6.60665796	3.46572077	Al
atom	3.72893053	16.51716577	3.36566893	Si
atom	5.39695781	6.40784425	3.70112732	Si
atom	14.69189455	16.66911435	3.27888731	Si
atom	1.47516845	7.85143565	1.85588718	Si
atom	10.79932617	18.09974575	1.74474563	Si
atom	16.53934623	7.82416492	1.91277117	Si
atom	7.65875231	18.08399423	2.00490662	Si
atom	1.66936120	12.85297655	5.59019672	Si
atom	10.56359943	2.53388942	5.58191320	Si
atom	16.87463375	12.82229892	5.59971154	Si
atom	7.44437074	2.54835617	5.75097384	Si
atom	1.57706657	4.74668961	1.93427219	Si
atom	10.78478141	14.85241864	1.75937227	Si
atom	16.58923067	4.69906065	1.99269745	Si
atom	7.65030177	14.92859231	1.92853045	Si
atom	1.57881470	16.05446356	5.50786347	Si
atom	10.59649405	5.70649433	5.49162548	Si
atom	16.73600232	16.03243129	5.55676791	Si
atom	7.52999355	5.78583269	5.85777180	Si

atom 10.37620802 6.28699297 2.44182500 Cu
atom 7.88414588 6.28125619 1.54571465 Cu
atom 8.74819219 6.29892673 3.04077569 O

Cartesian coordinates of the copper oxide cluster site in faujasite:

lattice_vector 24.3449999999999989 0.0000000000000000 0.0000000000000000
lattice_vector 0.0000000000000015 24.3449999999999989 0.0000000000000000
lattice_vector 0.0000000000000015 0.0000000000000015 24.3449999999999989
atom 21.8083 2.5367 0.0000 O
atom 21.8083 14.7092 12.1725 O
atom 9.6358 14.7092 0.0000 O
atom 9.6358 2.5367 12.1725 O
atom 0.0000 21.8083 2.5367 O
atom 0.0000 9.6358 14.7092 O
atom 12.1725 9.6358 2.5367 O
atom 12.1725 21.8083 14.7092 O
atom 2.5367 0.0000 21.8083 O
atom 2.5367 12.1725 9.6358 O
atom 14.7092 12.1725 21.8083 O
atom 14.7092 0.0000 9.6358 O
atom 2.5367 8.6230 6.0863 O
atom 2.5367 20.7955 18.2588 O
atom 14.7092 20.7955 6.0863 O
atom 14.8429 8.7126 18.2910 O
atom 0.0000 3.5495 8.6230 O
atom 0.0000 15.7220 20.7955 O
atom 12.1725 15.7220 8.6230 O
atom 12.1725 3.5495 20.7955 O
atom 21.8083 6.0863 3.5495 O
atom 21.8083 18.2588 15.7220 O
atom 9.6358 18.2588 3.5495 O
atom 9.6358 6.0863 15.7220 O
atom 3.5495 21.8083 6.0863 O
atom 3.5495 9.6358 18.2588 O
atom 15.7220 9.6358 6.0863 O
atom 15.7220 21.8083 18.2588 O
atom 6.0863 2.5367 8.6230 O
atom 6.0863 14.7092 20.7955 O
atom 18.2588 14.7092 8.6230 O
atom 18.2588 2.5367 20.7955 O
atom 8.6230 0.0000 3.5495 O
atom 8.6230 12.1725 15.7220 O
atom 20.7955 12.1725 3.5495 O
atom 20.7955 0.0000 15.7220 O
atom 8.6230 15.7220 12.1725 O
atom 8.6230 3.5495 0.0000 O
atom 20.7955 3.5495 12.1725 O
atom 20.7955 15.7220 0.0000 O
atom 6.0863 20.7955 14.7092 O

atom	6.0863	8.6230	2.5367	O
atom	18.2588	8.6230	14.7092	O
atom	18.2588	20.7955	2.5367	O
atom	3.5495	18.2588	9.6358	O
atom	3.5495	6.0863	21.8083	O
atom	15.7220	6.0863	9.6358	O
atom	15.7220	18.2588	21.8083	O
atom	2.5367	21.8083	0.0000	O
atom	2.5367	9.6358	12.1725	O
atom	14.7092	9.6358	0.0000	O
atom	14.7092	21.8083	12.1725	O
atom	21.8083	0.0000	2.5367	O
atom	21.8083	12.1725	14.7092	O
atom	9.6358	12.1725	2.5367	O
atom	9.6358	0.0000	14.7092	O
atom	0.0000	2.5367	21.8083	O
atom	0.0000	14.7092	9.6358	O
atom	12.1725	14.7092	21.8083	O
atom	12.1725	2.5367	9.6358	O
atom	8.6230	2.5367	6.0863	O
atom	8.6230	14.7092	18.2588	O
atom	20.7955	14.7092	6.0863	O
atom	20.7955	2.5367	18.2588	O
atom	3.5495	0.0000	8.6230	O
atom	3.5495	12.1725	20.7955	O
atom	15.7220	12.1725	8.6230	O
atom	15.7220	0.0000	20.7955	O
atom	6.0863	21.8083	3.5495	O
atom	6.0863	9.6358	15.7220	O
atom	18.2588	9.6358	3.5495	O
atom	18.2588	21.8083	15.7220	O
atom	21.8083	3.5495	6.0863	O
atom	21.7338	15.5869	18.2125	O
atom	9.6358	15.7220	6.0863	O
atom	9.6358	3.5495	18.2588	O
atom	2.5367	6.0863	8.6230	O
atom	2.5367	18.2588	20.7955	O
atom	14.7092	18.2588	8.6230	O
atom	14.7092	6.0863	20.7955	O
atom	0.0000	8.6230	3.5495	O
atom	0.0000	20.7955	15.7220	O
atom	12.1725	20.7955	3.5495	O
atom	12.1725	8.6230	15.7220	O
atom	15.7220	8.6230	12.1725	O
atom	15.7220	20.7955	0.0000	O
atom	3.5495	20.7955	12.1725	O
atom	3.5495	8.6230	0.0000	O
atom	20.7955	6.0863	14.7092	O
atom	20.7955	18.2588	2.5367	O
atom	8.6230	18.2588	14.7092	O

atom	8.6230	6.0863	2.5367	O
atom	18.2588	3.5495	9.6358	O
atom	18.2588	15.7220	21.8083	O
atom	6.0863	15.7220	9.6358	O
atom	6.0863	3.5495	21.8083	O
atom	23.5392	1.8551	1.8551	O
atom	23.5392	14.0276	14.0276	O
atom	11.3667	14.0276	1.8551	O
atom	11.3667	1.8551	14.0276	O
atom	1.8551	23.5392	1.8551	O
atom	1.8551	11.3667	14.0276	O
atom	14.0276	11.3667	1.8551	O
atom	14.0276	23.5392	14.0276	O
atom	1.8551	1.8551	23.5392	O
atom	1.8551	14.0276	11.3667	O
atom	14.0276	14.0276	23.5392	O
atom	14.0276	1.8551	11.3667	O
atom	0.8058	7.9413	7.9413	O
atom	0.8058	20.1138	20.1138	O
atom	12.9783	20.1138	7.9413	O
atom	12.9783	7.9413	20.1138	O
atom	22.4899	5.2804	7.9413	O
atom	22.4899	17.4529	20.1138	O
atom	10.3174	17.4529	7.9413	O
atom	10.3174	5.2804	20.1138	O
atom	22.4899	7.9413	5.2804	O
atom	22.4899	20.1138	17.4529	O
atom	10.3174	20.1138	5.2804	O
atom	10.3174	7.9413	17.4529	O
atom	5.2804	22.4899	7.9413	O
atom	5.2804	10.3174	20.1138	O
atom	17.4529	10.3174	7.9413	O
atom	17.4529	22.4899	20.1138	O
atom	7.9413	0.8058	7.9413	O
atom	7.9413	12.9783	20.1138	O
atom	20.1138	12.9783	7.9413	O
atom	20.1138	0.8058	20.1138	O
atom	7.9413	22.4899	5.2804	O
atom	7.9413	10.3174	17.4529	O
atom	20.1138	10.3174	5.2804	O
atom	20.1138	22.4899	17.4529	O
atom	6.8921	16.4037	14.0276	O
atom	6.8921	4.2312	1.8551	O
atom	19.0646	4.2312	14.0276	O
atom	19.0646	16.4037	1.8551	O
atom	4.2312	19.0646	14.0276	O
atom	4.2312	6.8921	1.8551	O
atom	16.4037	6.8921	14.0276	O
atom	16.4037	19.0646	1.8551	O
atom	4.2312	16.4037	11.3667	O

atom	4.2312	4.2312	23.5392	O
atom	16.4037	4.2312	11.3667	O
atom	16.4037	16.4037	23.5392	O
atom	0.8058	22.4899	22.4899	O
atom	0.8058	10.3174	10.3174	O
atom	12.9783	10.3174	22.4899	O
atom	12.9783	22.4899	10.3174	O
atom	22.4899	0.8058	22.4899	O
atom	22.4899	12.9783	10.3174	O
atom	10.3174	12.9783	22.4899	O
atom	10.3174	0.8058	10.3174	O
atom	22.4899	22.4899	0.8058	O
atom	22.4899	10.3174	12.9783	O
atom	10.3174	10.3174	0.8058	O
atom	10.3174	22.4899	12.9783	O
atom	23.5392	16.4037	16.4037	O
atom	23.5392	4.2312	4.2312	O
atom	11.3667	4.2312	16.4037	O
atom	11.3667	16.4037	4.2312	O
atom	1.8551	19.0646	16.4037	O
atom	1.8551	6.8921	4.2312	O
atom	14.0276	6.8921	16.4037	O
atom	14.0276	19.0646	4.2312	O
atom	1.8551	16.4037	19.0646	O
atom	1.8551	4.2312	6.8921	O
atom	14.0276	4.2312	19.0646	O
atom	14.0276	16.4037	6.8921	O
atom	19.0646	1.8551	16.4037	O
atom	19.0646	14.0276	4.2312	O
atom	6.8921	14.0276	16.4037	O
atom	6.8921	1.8551	4.2312	O
atom	16.4037	23.5392	16.4037	O
atom	16.4037	11.3667	4.2312	O
atom	4.2312	11.3667	16.4037	O
atom	4.2312	23.5392	4.2312	O
atom	16.4037	1.8551	19.0646	O
atom	16.4037	14.0276	6.8921	O
atom	4.2312	14.0276	19.0646	O
atom	4.2312	1.8551	6.8921	O
atom	17.4529	7.9413	10.3174	O
atom	17.4529	20.1138	22.4899	O
atom	5.2804	20.1138	10.3174	O
atom	5.2804	7.9413	22.4899	O
atom	20.1138	5.2804	10.3174	O
atom	20.1138	17.4529	22.4899	O
atom	7.9413	17.4529	10.3174	O
atom	7.9413	5.2804	22.4899	O
atom	20.1138	7.9413	12.9783	O
atom	20.1138	20.1138	0.8058	O
atom	7.9413	20.1138	12.9783	O

atom	7.9413	7.9413	0.8058	O
atom	24.2622	3.4789	24.2622	O
atom	24.2622	15.6514	12.0897	O
atom	12.0897	15.6514	24.2622	O
atom	12.0897	3.4789	12.0897	O
atom	24.2622	24.2622	3.4789	O
atom	24.2622	12.0897	15.6514	O
atom	12.0897	12.0897	3.4789	O
atom	12.0897	24.2622	15.6514	O
atom	3.4789	24.2622	24.2622	O
atom	3.4789	12.0897	12.0897	O
atom	15.6514	12.0897	24.2622	O
atom	15.6514	24.2622	12.0897	O
atom	0.0828	9.5652	6.0035	O
atom	0.0828	21.7377	18.1760	O
atom	12.2553	21.7377	6.0035	O
atom	12.2553	9.5652	18.1760	O
atom	0.0828	6.0035	9.5652	O
atom	0.0828	18.1760	21.7377	O
atom	12.2553	18.1760	9.5652	O
atom	12.2553	6.0035	21.7377	O
atom	20.8661	6.0035	6.0035	O
atom	20.8661	18.1760	18.1760	O
atom	8.6936	18.1760	6.0035	O
atom	8.6936	6.0035	18.1760	O
atom	6.0035	20.8661	6.0035	O
atom	6.0035	8.6936	18.1760	O
atom	18.1760	8.6936	6.0035	O
atom	18.1760	20.8661	18.1760	O
atom	6.0035	0.0828	9.5652	O
atom	6.0035	12.2553	21.7377	O
atom	18.1760	12.2553	9.5652	O
atom	18.1760	0.0828	21.7377	O
atom	9.5652	0.0828	6.0035	O
atom	9.5652	12.2553	18.1760	O
atom	21.7377	12.2553	6.0035	O
atom	21.7377	0.0828	18.1760	O
atom	6.1690	14.7799	12.0897	O
atom	6.1690	2.6073	24.2622	O
atom	18.3415	2.6073	12.0897	O
atom	18.3415	14.7799	24.2622	O
atom	6.1690	18.3415	15.6514	O
atom	6.1690	6.1690	3.4789	O
atom	18.3415	6.1690	15.6514	O
atom	18.3415	18.3415	3.4789	O
atom	2.6073	18.3415	12.0897	O
atom	2.6073	6.1690	24.2622	O
atom	14.7799	6.1690	12.0897	O
atom	14.7799	18.3415	24.2622	O
atom	0.0828	20.8661	0.0828	O

atom	0.0828	8.6936	12.2553	O
atom	12.2553	8.6936	0.0828	O
atom	12.2553	20.8661	12.2553	O
atom	0.0828	0.0828	20.8661	O
atom	0.0828	12.2553	8.6936	O
atom	12.2553	12.2553	20.8661	O
atom	12.2553	0.0828	8.6936	O
atom	20.8661	0.0828	0.0828	O
atom	20.8661	12.2553	12.2553	O
atom	8.6936	12.2553	0.0828	O
atom	8.6936	0.0828	12.2553	O
atom	24.2622	14.7799	18.3415	O
atom	24.2622	2.6073	6.1690	O
atom	12.0897	2.6073	18.3415	O
atom	12.0897	14.7799	6.1690	O
atom	24.2622	18.3415	14.7799	O
atom	24.2622	6.1690	2.6073	O
atom	12.0897	6.1690	14.7799	O
atom	12.0897	18.3415	2.6073	O
atom	3.4789	18.3415	18.3415	O
atom	3.4789	6.1690	6.1690	O
atom	15.6514	6.1690	18.3415	O
atom	15.6514	18.3415	6.1690	O
atom	18.3415	3.4789	18.3415	O
atom	18.3415	15.6514	6.1690	O
atom	6.1690	15.6514	18.3415	O
atom	6.1690	3.4789	6.1690	O
atom	18.3415	24.2622	14.7799	O
atom	18.3415	12.0897	2.6073	O
atom	6.1690	12.0897	14.7799	O
atom	6.1690	24.2622	2.6073	O
atom	14.7799	24.2622	18.3415	O
atom	14.7799	12.0897	6.1690	O
atom	2.6073	12.0897	18.3415	O
atom	2.6073	24.2622	6.1690	O
atom	18.1760	9.5652	12.2553	O
atom	18.1760	21.7377	0.0828	O
atom	6.0035	21.7377	12.2553	O
atom	6.0035	9.5652	0.0828	O
atom	18.1760	6.0035	8.6936	O
atom	18.1760	18.1760	20.8661	O
atom	6.0035	18.1760	8.6936	O
atom	6.0035	6.0035	20.8661	O
atom	21.7377	6.0035	12.2553	O
atom	21.7377	18.1760	0.0828	O
atom	9.5652	18.1760	12.2553	O
atom	9.5652	6.0035	0.0828	O
atom	22.5995	4.3091	1.7772	O
atom	22.5995	16.4816	13.9497	O
atom	10.4270	16.4816	1.7772	O

atom	10.4270	4.3091	13.9497	O
atom	1.7772	22.5995	4.3091	O
atom	1.7772	10.4270	16.4816	O
atom	13.9497	10.4270	4.3091	O
atom	13.9497	22.5995	16.4816	O
atom	4.3091	1.7772	22.5995	O
atom	4.3091	13.9497	10.4270	O
atom	16.4796	13.8525	22.4827	O
atom	16.4816	1.7772	10.4270	O
atom	1.7455	10.3953	7.8634	O
atom	1.7455	22.5678	20.0359	O
atom	13.9180	22.5678	7.8634	O
atom	14.0144	10.4531	20.0486	O
atom	22.5678	4.3407	10.3953	O
atom	22.5678	16.5132	22.5678	O
atom	10.3953	16.5132	10.3953	O
atom	10.3953	4.3407	22.5678	O
atom	20.0359	7.8634	4.3407	O
atom	20.0359	20.0359	16.5132	O
atom	7.8634	20.0359	4.3407	O
atom	7.8634	7.8634	16.5132	O
atom	4.3407	20.0359	7.8634	O
atom	4.3407	7.8634	20.0359	O
atom	16.5132	7.8634	7.8634	O
atom	16.5132	20.0359	20.0359	O
atom	7.8634	1.7455	10.3953	O
atom	7.8634	13.9180	22.5678	O
atom	20.0359	13.9180	10.3953	O
atom	20.0359	1.7455	22.5678	O
atom	10.3953	22.5678	4.3407	O
atom	10.3953	10.3953	16.5132	O
atom	22.5678	10.3953	4.3407	O
atom	22.5678	22.5678	16.5132	O
atom	7.8318	13.9497	13.9497	O
atom	7.8318	1.7772	1.7772	O
atom	20.0043	1.7772	13.9497	O
atom	20.0043	13.9497	1.7772	O
atom	4.3091	20.0043	16.4816	O
atom	4.3091	7.8318	4.3091	O
atom	16.4816	7.8318	16.4816	O
atom	16.4816	20.0043	4.3091	O
atom	1.7772	16.4816	10.4270	O
atom	1.7772	4.3091	22.5995	O
atom	13.9497	4.3091	10.4270	O
atom	13.9497	16.4816	22.5995	O
atom	4.3091	22.5995	1.7772	O
atom	4.3091	10.4270	13.9497	O
atom	16.4816	10.4270	1.7772	O
atom	16.4816	22.5995	13.9497	O
atom	22.5995	1.7772	4.3091	O

atom	22.4532	13.8888	16.5935	O
atom	10.4270	13.9497	4.3091	O
atom	10.4270	1.7772	16.4816	O
atom	10.3953	1.7455	7.8634	O
atom	10.3953	13.9180	20.0359	O
atom	22.5678	13.9180	7.8634	O
atom	22.5678	1.7455	20.0359	O
atom	4.3407	22.5678	10.3953	O
atom	4.3407	10.3953	22.5678	O
atom	16.5132	10.3953	10.3953	O
atom	16.5132	22.5678	22.5678	O
atom	20.0359	4.3407	7.8634	O
atom	20.0359	16.5132	20.0359	O
atom	7.8634	16.5132	7.8634	O
atom	7.8634	4.3407	20.0359	O
atom	1.7455	7.8634	10.3953	O
atom	1.7455	20.0359	22.5678	O
atom	13.9180	20.0359	10.3953	O
atom	13.9180	7.8634	22.5678	O
atom	13.9497	7.8318	13.9497	O
atom	13.9497	20.0043	1.7772	O
atom	1.7772	20.0043	13.9497	O
atom	1.7772	7.8318	1.7772	O
atom	20.0043	4.3091	16.4816	O
atom	20.0043	16.4816	4.3091	O
atom	7.8318	16.4816	16.4816	O
atom	7.8318	4.3091	4.3091	O
atom	20.0359	22.5678	1.7455	O
atom	20.0611	10.3404	14.0443	O
atom	7.8634	10.3953	1.7455	O
atom	7.8634	22.5678	13.9180	O
atom	4.3091	16.4816	20.0043	O
atom	4.3091	4.3091	7.8318	O
atom	16.4816	4.3091	20.0043	O
atom	16.4816	16.4816	7.8318	O
atom	13.9497	1.7772	20.0043	O
atom	13.9497	13.9497	7.8318	O
atom	1.7772	13.9497	20.0043	O
atom	1.7772	1.7772	7.8318	O
atom	22.5678	7.8634	13.9180	O
atom	22.5678	20.0359	1.7455	O
atom	10.3953	20.0359	13.9180	O
atom	10.3953	7.8634	1.7455	O
atom	23.0523	3.0456	0.8862	Si
atom	23.0523	15.2181	13.0587	Si
atom	10.8798	15.2181	0.8862	Si
atom	10.8798	3.0456	13.0587	Si
atom	0.8862	23.0523	3.0456	Si
atom	0.8862	10.8798	15.2181	Si
atom	13.0587	10.8798	3.0456	Si

atom	13.0587	23.0523	15.2181	Si
atom	3.0456	0.8862	23.0523	Si
atom	3.0456	13.0587	10.8798	Si
atom	15.3478	13.0096	22.7933	Al
atom	15.2181	0.8862	10.8798	Si
atom	1.2927	9.1318	6.9724	Si
atom	1.2927	21.3043	19.1449	Si
atom	13.4652	21.3043	6.9724	Si
atom	13.8304	9.0837	19.3689	Al
atom	23.4588	4.7935	9.1318	Si
atom	23.4588	16.9660	21.3043	Si
atom	11.2863	16.9660	9.1318	Si
atom	11.2863	4.7935	21.3043	Si
atom	21.2994	6.9724	4.7935	Si
atom	21.2994	19.1449	16.9660	Si
atom	9.1269	19.1449	4.7935	Si
atom	9.1269	6.9724	16.9660	Si
atom	4.7935	21.2994	6.9724	Si
atom	4.7935	9.1269	19.1449	Si
atom	16.9660	9.1269	6.9724	Si
atom	16.9660	21.2994	19.1449	Si
atom	6.9724	1.2927	9.1318	Si
atom	6.9724	13.4652	21.3043	Si
atom	19.1449	13.4652	9.1318	Si
atom	19.1449	1.2927	21.3043	Si
atom	9.1318	23.4588	4.7935	Si
atom	9.1318	11.2863	16.9660	Si
atom	21.3043	11.2863	4.7935	Si
atom	21.3043	23.4588	16.9660	Si
atom	7.3790	15.2132	13.0587	Si
atom	7.3790	3.0407	0.8862	Si
atom	19.5515	3.0407	13.0587	Si
atom	19.5515	15.2132	0.8862	Si
atom	5.2001	19.5515	15.2181	Si
atom	5.2001	7.3790	3.0456	Si
atom	17.3726	7.3790	15.2181	Si
atom	17.3726	19.5515	3.0456	Si
atom	3.0407	17.3726	10.8798	Si
atom	3.0407	5.2001	23.0523	Si
atom	15.2132	5.2001	10.8798	Si
atom	15.2132	17.3726	23.0523	Si
atom	3.0456	23.0523	0.8862	Si
atom	3.0456	10.8798	13.0587	Si
atom	15.2181	10.8798	0.8862	Si
atom	15.2181	23.0523	13.0587	Si
atom	23.0523	0.8862	3.0456	Si
atom	23.0523	13.0587	15.2181	Si
atom	10.8798	13.0587	3.0456	Si
atom	10.8798	0.8862	15.2181	Si
atom	0.8862	3.0456	23.0523	Si

atom	0.8862	15.2181	10.8798	Si
atom	13.0587	15.2181	23.0523	Si
atom	13.0587	3.0456	10.8798	Si
atom	9.1318	1.2927	6.9724	Si
atom	9.1318	13.4652	19.1449	Si
atom	21.3043	13.4652	6.9724	Si
atom	21.3043	1.2927	19.1449	Si
atom	4.7935	23.4588	9.1318	Si
atom	4.7935	11.2863	21.3043	Si
atom	16.9660	11.2863	9.1318	Si
atom	16.9660	23.4588	21.3043	Si
atom	6.9724	21.2994	4.7935	Si
atom	6.9724	9.1269	16.9660	Si
atom	19.1449	9.1269	4.7935	Si
atom	19.1449	21.2994	16.9660	Si
atom	21.2994	4.7935	6.9724	Si
atom	21.2994	16.9660	19.1449	Si
atom	9.1269	16.9660	6.9724	Si
atom	9.1269	4.7935	19.1449	Si
atom	1.2927	6.9724	9.1318	Si
atom	1.2927	19.1449	21.3043	Si
atom	13.4652	19.1449	9.1318	Si
atom	13.4652	6.9724	21.3043	Si
atom	23.4588	9.1318	4.7935	Si
atom	23.4588	21.3043	16.9660	Si
atom	11.2863	21.3043	4.7935	Si
atom	11.2863	9.1318	16.9660	Si
atom	15.2132	7.3790	13.0587	Si
atom	15.2132	19.5515	0.8862	Si
atom	3.0407	19.5515	13.0587	Si
atom	3.0407	7.3790	0.8862	Si
atom	19.5515	5.2001	15.2181	Si
atom	19.5515	17.3726	3.0456	Si
atom	7.3790	17.3726	15.2181	Si
atom	7.3790	5.2001	3.0456	Si
atom	17.3726	3.0407	10.8798	Si
atom	17.3726	15.2132	23.0523	Si
atom	5.2001	15.2132	10.8798	Si
atom	5.2001	3.0407	23.0523	Si
atom	1.2927	21.2994	23.4588	Si
atom	1.2927	9.1269	11.2863	Si
atom	13.4652	9.1269	23.4588	Si
atom	13.4652	21.2994	11.2863	Si
atom	23.4588	1.2927	21.2994	Si
atom	23.4588	13.4652	9.1269	Si
atom	11.2863	13.4652	21.2994	Si
atom	11.2863	1.2927	9.1269	Si
atom	21.2994	23.4588	1.2927	Si
atom	21.1025	11.2039	13.5272	Al
atom	9.1269	11.2863	1.2927	Si

atom	9.1269	23.4588	13.4652	Si
atom	22.8450	15.0439	17.3951	Al
atom	23.0523	3.0407	5.2001	Si
atom	10.8798	3.0407	17.3726	Si
atom	10.8798	15.2132	5.2001	Si
atom	0.8862	19.5515	15.2132	Si
atom	0.8862	7.3790	3.0407	Si
atom	13.0587	7.3790	15.2132	Si
atom	13.0587	19.5515	3.0407	Si
atom	3.0456	17.3726	19.5515	Si
atom	3.0456	5.2001	7.3790	Si
atom	15.2181	5.2001	19.5515	Si
atom	15.2181	17.3726	7.3790	Si
atom	19.5515	3.0456	17.3726	Si
atom	19.5515	15.2181	5.2001	Si
atom	7.3790	15.2181	17.3726	Si
atom	7.3790	3.0456	5.2001	Si
atom	17.3726	23.0523	15.2132	Si
atom	17.3726	10.8798	3.0407	Si
atom	5.2001	10.8798	15.2132	Si
atom	5.2001	23.0523	3.0407	Si
atom	15.2132	0.8862	19.5515	Si
atom	15.2132	13.0587	7.3790	Si
atom	3.0407	13.0587	19.5515	Si
atom	3.0407	0.8862	7.3790	Si
atom	16.9660	9.1318	11.2863	Si
atom	16.9660	21.3043	23.4588	Si
atom	4.7935	21.3043	11.2863	Si
atom	4.7935	9.1318	23.4588	Si
atom	19.1449	4.7935	9.1269	Si
atom	19.1449	16.9660	21.2994	Si
atom	6.9724	16.9660	9.1269	Si
atom	6.9724	4.7935	21.2994	Si
atom	21.3043	6.9724	13.4652	Si
atom	21.3043	19.1449	1.2927	Si
atom	9.1318	19.1449	13.4652	Si
atom	9.1318	6.9724	1.2927	Si
atom	21.2994	1.2927	23.4588	Si
atom	21.2994	13.4652	11.2863	Si
atom	9.1269	13.4652	23.4588	Si
atom	9.1269	1.2927	11.2863	Si
atom	1.2927	23.4588	21.2994	Si
atom	1.2927	11.2863	9.1269	Si
atom	13.4652	11.2863	21.2994	Si
atom	13.4652	23.4588	9.1269	Si
atom	23.4588	21.2994	1.2927	Si
atom	23.4588	9.1269	13.4652	Si
atom	11.2863	9.1269	1.2927	Si
atom	11.2863	21.2994	13.4652	Si
atom	15.2132	23.0523	17.3726	Si

atom	15.2132	10.8798	5.2001	Si
atom	3.0407	10.8798	17.3726	Si
atom	3.0407	23.0523	5.2001	Si
atom	19.5515	0.8862	15.2132	Si
atom	19.5515	13.0587	3.0407	Si
atom	7.3790	13.0587	15.2132	Si
atom	7.3790	0.8862	3.0407	Si
atom	17.3726	3.0456	19.5515	Si
atom	17.3726	15.2181	7.3790	Si
atom	5.2001	15.2181	19.5515	Si
atom	5.2001	3.0456	7.3790	Si
atom	3.0456	19.5515	17.3726	Si
atom	3.0456	7.3790	5.2001	Si
atom	15.2181	7.3790	17.3726	Si
atom	15.2181	19.5515	5.2001	Si
atom	23.0523	17.3726	15.2132	Si
atom	23.0523	5.2001	3.0407	Si
atom	10.8798	5.2001	15.2132	Si
atom	10.8798	17.3726	3.0407	Si
atom	0.8862	15.2132	19.5515	Si
atom	0.8862	3.0407	7.3790	Si
atom	13.0587	3.0407	19.5515	Si
atom	13.0587	15.2132	7.3790	Si
atom	9.1318	16.9660	11.2863	Si
atom	9.1318	4.7935	23.4588	Si
atom	21.3043	4.7935	11.2863	Si
atom	21.3043	16.9660	23.4588	Si
atom	4.7935	19.1449	9.1269	Si
atom	4.7935	6.9724	21.2994	Si
atom	16.9660	6.9724	9.1269	Si
atom	16.9660	19.1449	21.2994	Si
atom	6.9724	21.3043	13.4652	Si
atom	6.9724	9.1318	1.2927	Si
atom	19.1449	9.1318	13.4652	Si
atom	19.1449	21.3043	1.2927	Si
atom	17.6940	10.0318	16.3417	Cu
atom	17.4170	12.9302	17.4845	Cu
atom	16.6886	12.5160	20.4980	Cu
atom	17.5655	10.8558	19.9738	O
atom	17.0521	13.8757	19.1388	O
atom	16.1672	10.3312	18.7355	Cu
atom	19.1339	11.2539	18.9019	Cu
atom	17.9803	9.8312	18.2597	O
atom	18.7822	14.2250	19.9831	Cu
atom	17.8407	11.9272	15.8789	O
atom	20.4281	13.8093	17.6876	Cu
atom	20.0637	12.8949	19.3594	O
atom	19.7877	11.7408	15.8268	Cu
atom	20.4784	11.8704	17.6453	O

References

S1. V. Blum, R. Gehrke, F. Hanke, P. Havu, V. Havu, X. Ren, K. Reuter, M. Scheffler, *Comput. Phys. Commun.* **2009**, *180*, 2175-2196.

S2. X. Ren, P. Rinke, V. Blum, J.Wieferink, A. Tkatchenko, A. Sanfilippo, K. Reuter, M. Scheffler, *New J. Phys.* **2012**, *14*, 053020.

S3. C. Adamo, V. Barone, *J. Chem. Phys.* **1999**, *110*, 6158-6170.

S4. A. Tkatchenko, M. Scheffler, *Phys. Rev. Lett.* **2009**, *102*, 073005.
